# Phonetic and Phono-Lexical Accuracy of Non-Native Tone Production by English-L1 and Mandarin-L1 Speakers

**DOI:** 10.1177/00238309221143719

**Published:** 2023-01-15

**Authors:** Tim Joris Laméris, Katrina Kechun Li, Brechtje Post

**Affiliations:** Phonetics Laboratory, University of Cambridge, UK

**Keywords:** Production, lexical tone, L2, word learning, individual variability

## Abstract

Lexical tones are known to be a challenging aspect of speech to acquire in a second language, but several factors are known to affect tone learning facility, such as L1 tonal status (whether a learner’s L1 is tonal or not), tone type (the shape of the tones to be acquired), and individual extralinguistic factors (such as musicianship, pitch aptitude, and working memory). Crucially, most of our knowledge of the effect of these factors is based on evidence from perception. The production side of tone learning and the origins of individual variability in learning facility remain relatively understudied. To this end, this study investigated non-native tone production—both in terms of phonetic accuracy in a pseudoword imitation task and in terms of phono-lexical accuracy in a picture-naming task—by English-L1 and Mandarin-L1 speakers. Results show that L1 tonal status and tone type dynamically affected both imitation and picture-naming accuracy, as there were specific accuracy patterns for the English and Mandarin groups. Production accuracy was further facilitated by individual musical experience, working memory, and pitch aptitude. This study’s findings add to the currently limited literature on how both language-specific and individual extralinguistic factors modulate non-native tone processing in the speaking modality.

## 1 Introduction

Lexical tones are a relatively difficult aspect of speech to acquire in a second language (L2) for adult learners.^1^ Although learners may overcome difficulties in processing tones devoid of word meaning, for instance, in tone identification or discrimination tasks (Tsukada & Kondo, 2019; X. Wang, 2013), it appears that the processing of tones at a lexical level, for instance, in word identification tasks, is particularly challenging (Ling & Grüter, 2022; Pelzl et al., 2019, 2021a, 2021b). Yet, some individuals learn tones more easily than others do, and previous studies have shown that tone learning facility may be guided by individual factors such as L1 tonal status, which refers to the functionality of pitch in the L1 (Burnham et al., 2015; Laméris, 2022; Schaefer & Darcy, 2014), but also tone type, which refers to the shape of F0-based units (either tonal or intonational) in the learner’s L1 and their similarity to L2 target tones (Chen et al., 2020; Hao, 2012; So & Best, 2010; K. Yu et al., 2017). In addition, extralinguistic factors such as musical experience, pitch perception aptitude, and working memory (WM) have been found to influence the ease with which individuals process non-native tones (Bowles et al., 2016; Laméris & Post, 2022; Perrachione et al., 2011; Wong & Perrachione, 2007).

Crucially, most of our knowledge of individual variability in L2 tone learning comes from studies that target the listening modality (i.e., perception). Tone learning in the speaking modality (i.e., production) and the individual factors that affect it have received much less attention, as will be discussed in Section 2. To provide a more complete account of non-native tone processing, we therefore investigated the combined effects of individual factors on non-native production. In particular, we ask how L1 tonal status, tone type, and extralinguistic factors affect tone production *facility*, which refers to the ease with which individuals process non-native sounds in the earliest stages of encountering a non-native system (Bowles et al., 2016, p. 775). To this end, we observed non-native tone production of tonal pseudowords by English-L1 and Mandarin-L1 speakers. We measured their *phonetic accuracy*, which here refers to the ability to produce a tone in fine-grained phonetic terms with reference to a target production. We also measured their *phono-lexical accuracy*, which here refers to the ability to link a phonological tone category to a lexical representation. We employed an imitation task to measure phonetic accuracy, and a picture-naming task to measure phono-lexical accuracy.

An imitation task can be described as “a production task adopting auditory instead of orthographic prompts” (Hao & de Jong, 2016, p. 156) in which upon presentation of an auditory target stimulus, speakers are asked to repeat that stimulus out loud and as accurately as possible. An imitation task was deemed to be a suitable tool to investigate individual differences in phonetic accuracy in tone production. This is because individuals are expected to perform relatively well in imitation tasks, and mostly differ between one another in terms of measurable F0-based values rather than in overt phonological categories (Dong et al., 2019; Hao, 2012). For instance, participants who hear a pseudoword stimulus /lɔn/ with a rising tone are all expected to accurately reproduce that stimulus with a rising pitch movement, but the fine-grained acoustic nature of that pitch movement relative to the target stimulus (i.e., the phonetic accuracy) may differ between participants. The first aim of this article is to investigate whether individual differences in phonetic accuracy of non-native tone production can be attributed to an individual’s L1 tonal status, tone type, and extralinguistic factors. We investigated this by examining the properties of participants’ F0 trajectories (using discrete cosine transformation [DCT], see Section 3) and the tonal distance relative to the target.

Although imitation tasks may reveal part of the production side of tone learning, real-life L2 learners rarely have constant access to target sounds that they can then imitate. Instead, as active language users, they are likely to be involved in lexical production, which involves the breaking down of a lexical item into “sub-lexical representations” and subsequently into speech (Schmitz et al., 2018, p. 529). In this study, we operationalized lexical production by means of a picture-naming task, in which participants are presented with an image of a lexical item that they are then asked to name in an L2 (Barcroft & Sommers, 2014; Li & Dekeyser, 2017; A. Yu et al., 2022). We deemed a picture-naming task to be a suitable task to examine phono-lexical accuracy, as it requires a participant to retrieve a lexical representation and produce it with the correct tone category. Because it is the production of a correct phonological tone category to indicate lexical meaning that is of interest here, and not the phonetic finesse of that production per se, phono-lexical accuracy was determined via auditory labeling by two raters, following similar earlier studies (Dong et al., 2019; Hao, 2012). Thus, this article’s second aim is to investigate whether the ease with which some individuals retain and link tonal categories to lexical representations in the speaking modality is in any way modulated by L1 tonal status, tone type, and extralinguistic factors.

## 2 Background

### 2.1 Effects of L1 tonal status on non-native tone production

A pertinent question in the tone learning literature is whether the lexical functionality of pitch in the L1 (henceforth: “L1 tonal status”) affects non-native tone processing (Burnham et al., 2015; Laméris, 2022; Schaefer & Darcy, 2014). This question is driven by the intuition that, compared with speakers of a tonal language like Mandarin Chinese, speakers of a non-tone language like English may find it relatively difficult to learn lexical tones because “there is nothing in their native grammar that prepares them for using prosodic properties such as F0 in a lexically contrastive manner” (Francis et al., 2008, p. 269). Yet despite this intuition, there is mixed empirical evidence for a facilitative effect of L1 tonal status on non-native tone processing. Some studies show that tonal L1 speakers (henceforth: tonal L1ers) outperform non-tonal L1ers in non-native tone perception (Burnham et al., 2015; Chan & Leung, 2020; Schaefer & Darcy, 2014) and word learning (Poltrock et al., 2018). Yet, other studies show that L1 tonal status has no clear facilitative effect (Cooper & Wang, 2012; Francis et al., 2008; Gandour & Harshman, 1978; So & Best, 2010) or that it may even hinder non-native tone processing (Chiao et al., 2011; X. Wang, 2013). Although this discrepancy in findings may be due to methodological differences in terms of the languages and tone types involved and the level of processing probed by the task at hand (pre-lexical or lexical), it is crucial to note that all these studies investigated tone processing in the listening modality. There appear to be only a few studies that shed light on whether L1 tonal status facilitates non-native tone processing in the speaking modality, in terms of either phonetic or phono-lexical accuracy.

Why would L1 tonal status facilitate non-native tone production? The underlying intuition as to why this might be the case is similar to the intuition regarding L1 tonal status and perception. For instance, the Feature Hypothesis (although designed as a hypothesis for the non-native processing of duration) posits that “L2 features not used to signal phonological contrast in L1 will be difficult to perceive for the L2 learners and this difficulty will be reflected in the learner’s production (..)” (McAllister et al., 2002, p. 230).

Findings from several studies indeed suggest that speakers of non-tonal languages may have a relative difficulty in tone production. For instance, Y. Wang et al. (2003) evaluated phonetic accuracy in a read-aloud task of Mandarin words written in pinyin romanization by English-L1 elementary learners. An acoustic analysis between learners’ and native speakers’ productions showed that even though learners’ phonetic tone accuracy improved after a training session, their productions still deviated from native norms in terms of pitch height and pitch contour. Similarly, in a read-aloud task of Mandarin pinyin words by native speakers and German-L1 elementary learners, Ding et al. (2011) report that learners employed a narrower pitch range and produced less defined pitch changes than native Mandarin speakers. Kirby and Giang (2021) investigated the production of Vietnamese tones by speakers of Khmer Krom, a non-tonal Austroasiatic language. Their acoustic analyses revealed that in general, Khmer Krom productions had a more compressed pitch range and lacked clear distinctions between two complex contour tones in comparison to native Vietnamese productions.

These studies suggest that non-tonal L1ers may have difficulty in fully exploiting the phonetic properties of non-native tones. However, these studies all compared *non-native* tone production by *non-tonal* L1ers with *native* tone production by *tonal* L1ers. It is thus unclear whether the reported L2 learners’ phonetic accuracy is an effect of their L1 tonal status (i.e., their inexperience with tones leads to “poorer” L2 tone production) or a general effect of operating in a non-native system, which may naturally lead to production phenomena such as reduced pitch range (Grazia Busà & Urbani, 2011; Zimmerer et al., 2014).

To the best of our knowledge, there are only three cross-linguistic studies (Hao, 2012; Yang, 2019; A. Yu et al., 2022) that shed light on how tonal and non-tonal L1ers differ in tone production in a language that is non-native to both:

Hao (2012) investigated production of Mandarin tones in an imitation task and in a read-aloud task of Mandarin pinyin words by Cantonese-L1 and English-L1 intermediate learners of Mandarin. Based on production accuracy scores provided by native raters, Hao (2012) reports that Cantonese-L1 speakers’ production was overall not more accurate than that of English-L1 speakers.

Yang (2019) investigated phono-lexical tone production accuracy by means of a read-aloud task of Mandarin characters by speakers of Thai and Yoruba (both tonal languages) and found that Thai speakers outperformed Yoruba speakers. However, the Thai speakers had more exposure to Mandarin than did the Yoruba speakers, which may have influenced performance.

A. Yu et al. (2022) examined phonetic accuracy of Cantonese tone productions by Hong Kong Cantonese native speakers and L2 speakers who spoke Urdu, Punjabi, or English as their dominant language. Production was elicited by a picture-naming task, making the task phono-lexical in nature, but accuracy was measured phonetically by a DCT analysis, which yielded three coefficients (DCT1, DCT2, and DCT3) that respectively describe a tone production’s mean pitch height, slope, and curvature. In addition, they calculated the tonal distance between learners’ and native speakers’ productions, which can be taken as a measure of phonetic accuracy of tone productions relative to the native target production. The results of these analyses revealed no clear evidence that speakers of Punjabi (a tonal language) produced tones more accurately than did speakers of Urdu or English (both non-tonal languages).

In sum, findings from previous studies do not clearly show that tonal L1ers outperform non-tonal L1ers in tone production, either in their ability to accurately produce tones at a fine-grained level (i.e., their phonetic accuracy) or in their ability to retrieve and produce a specific phonological tone category that is linked to a specific lexical meaning (i.e., their phono-lexical accuracy).

### 2.2 Effects of L1 tone type on non-native tone production

The term “tone type” will be used henceforth as an overarching expression to describe the nature of F0-based units (which can be either lexical or intonational tones) in terms of (1) phonetic-acoustic and (2) phonological-categorical properties, following the distinction proposed by K. Yu et al. (2017). It is important to consider tone type because rather than an individual’s L1 tonal status alone, it is often the individual’s L1 tone types and their interaction with L2 tone types that determine how easily specific L2 tones are processed. Most of our knowledge of the effect of tone type comes from perception studies, but it is worth summarizing some of those findings here.

In terms of the phonetic-acoustic nature of tone type, it has been suggested that Mandarin speakers pay relatively more attention to differences in pitch contour and direction, whereas English speakers attune predominantly to pitch height differences. This may make the perception of L2 level tone contrasts relatively difficult for Mandarin speakers and relatively easy for English speakers (Francis et al., 2008; Gandour & Harshman, 1978; Qin & Jongman, 2016).

In terms of the phonological-categorical nature of tone type, it has been suggested that Mandarin speakers assimilate non-native tone categories to their most similar-sounding L1 tone categories (Chen et al., 2020; Hao, 2012; So & Best, 2010). This notion of categorical assimilation is rooted in theoretical models such as the Perceptual Assimilation Model (PAM) that propose that the ease with which non-native sounds are learned depends on the relative similarity between L1 and L2 sounds (Best, 1995; Best & Tyler, 2007). For instance, Mandarin-L1 speakers appear to struggle with the discrimination of Cantonese mid-level and low-level tones because they may assimilate Cantonese level tone categories to the Mandarin level tone in a two-to-one fashion (Qin & Jongman, 2016, p. 334; Zhu et al., 2021, p. 4224). Although speakers of English and other non-tonal languages may also assimilate non-native tones to intonational categories, it appears that the effect of such assimilation on L2 tone perception is relatively weak (Reid et al., 2015; So & Best, 2010), arguably because intonational categories have a “weaker (less categorical) mental representation” than lexical tone categories (Francis et al., 2008, p. 269). Instead, non-tonal L1ers may process non-native tones more psychoacoustically (Best, 2019, p. 5; K. Yu et al., 2019).

There are a few studies that show possible effects of tone type in non-native tone production. Indeed, the previously mentioned studies by Hao (2012) and A. Yu et al. (2022) observed both global and L1-specific patterns of production accuracy per tone type. Some of these L1-specific patterns were attributed to potential interference with L1 tone types. For instance, Hao (2012) observed that whereas both English-L1 and Cantonese-L1 speakers had difficulty in the accurate production of the Mandarin falling and dipping tones, Cantonese speakers in addition appeared to have struggled in the production of the Mandarin high-level tone. Hao suggests that the production of the falling and dipping tone may have been intrinsically difficult for both English and Cantonese speakers because of their acoustic similarity, whereas the additional difficulty of producing the level tone for Cantonese speakers may have been due to interference from Cantonese types (Hao, 2012, p. 278). However, A. Yu et al. (2022, p. 13) report that “there was remarkably little difference” in tone production across English-L1, Punjabi-L1, and Urdu-L1 speakers who all had less distinct productions of the two Cantonese rising tones and the three Cantonese level tones compared with native speakers. They allude to the notion that Punjabi-L1 speakers may have assimilated the mid-level and low-level tones to the Punjabi high-level tone, and show that the Punjabi-L1 speakers exhibited L1-specific performance in their perception, but this was not observed in their production.

More direct evidence for the effect of tone type on tone production comes from a read-aloud task of Cantonese words (romanized with tone marks) by native Cantonese and naïve Mandarin speakers by Zhang and Peng (2017). They suggest that mismatches between Mandarin and Cantonese tone inventories modulated phonetic production accuracy. Based on the relative acoustic distance of Mandarin productions from the native target, they showed that productions of the Cantonese high-level tone (which resembles the Mandarin high-level tone) deviated least from the target, whereas productions of mid-level and low-level tones (which have no equivalent in Mandarin) deviated more. These findings could be taken as evidence for an effect of tone type in production, as Mandarin speakers may pay less attention to pitch height differences at the phonetic level and/or assimilate Cantonese level contrasts to their native high-level tone at a phonological level.

### 2.3 Effects of extralinguistic factors on L2 tone production

To explain how individual performance in L2 tone production is modulated by not only language-specific factors such as L1 tonal status and tone type, but additionally by individual extralinguistic factors, we also investigated the effect of musical experience, pitch aptitude, and WM. There are a handful of studies that have separately investigated the effects of these factors on non-native tone production.

Musical experience refers to the years of musical practice an individual may have had, often through formal training. It is assumed to enhance pitch acuity in the domain of music, which may be transferred to the domain of speech (Choi, 2021; Patel, 2011). Studies on the effect of musical experience on tone production have shown that English-L1 individuals with musical experience are better than non-musicians at imitating Mandarin tones (Gottfried et al., 2004). Similarly, English-L1 speakers with enhanced musical sensitivity (measured by pitch change perception and memory tests) have been found to sound more native-like in Mandarin read-aloud and picture-naming tasks (Li & Dekeyser, 2017). We are not aware of any non-native tone production studies that have investigated the effect of musical experience for Mandarin speakers, and therefore, in this study we investigate the extent to which musical experience affects non-native tone production performance for both English and Mandarin speakers.

We also investigated the effect of pitch aptitude. Pitch aptitude here refers to the ability to perceive and categorize tones devoid of lexical meaning, as measured by a tone categorization task. A typical format of a tone categorization task is one where a participant listens to a monosyllabic auditory stimulus and is asked to identify the tone category, for instance, by selecting an image that represents the pitch contour (Bowles et al., 2016; Dong et al., 2019; Laméris & Post, 2022; Wong & Perrachione, 2007). Previous studies have referred to this particular measure using different terminologies, namely, “pitch identification” (Wong & Perrachione, 2007), “basic perceptual abilities for pitch” (Perrachione et al., 2011), “pitch ability” or “pitch processing” (Bowles et al., 2016), “individual aptitude” (Dong et al., 2019), or “phonological processing” (Ling & Grüter, 2022). For coherence, we will henceforth use the term “pitch aptitude” to refer to this measure. In a study by Dong et al. (2019), pitch aptitude (measured by a tone categorization task) significantly predicted performance in tone imitation, and marginally so for performance in a picture-naming task of Mandarin words by English-L1 naïve learners. However, Hao (2012) found that pitch aptitude did not, or did only weakly correlate with Mandarin tone imitation by English-L1 and Cantonese-L1 intermediate learners. Conversely, pitch aptitude moderately correlated with the read-aloud task (although some of the correlations were negative, suggesting that better perceptual skills were associated with worse production skills). Given this limited and somehow mixed evidence on the relationship between pre-lexical tone perception and tone production, we deemed it important to investigate the effect of pitch aptitude on tone imitation and picture-naming in the present study.

WM refers to the individual capacity to recall and process strings of information, as can be measured by a digit span task (Baddeley, 2003; Mattys & Baddeley, 2019). To the best of our knowledge, there are no studies that have investigated the effect of WM on non-native tone production, although there exist some studies on non-native tone perception and word learning in the listening modality. These studies show mixed evidence of a facilitative effect of WM: some studies suggest that WM facilitates non-native tone perception and word learning (Bowles et al., 2016; Goss, 2020; Ingvalson et al., 2017; Laméris & Post, 2022), whereas others do not find a clear link (Goss & Tamaoka, 2019; Perrachione et al., 2011). Although the strength of the effect of WM can depend heavily on the nature of the WM task and the specific aspect of non-native speech learning at hand (Bidelman et al., 2013; Hutka et al., 2015), WM is well known to have an important effect on second language processing, particularly in word learning (Atkins & Baddeley, 1998; Gupta, 2003; Kormos & Sáfár, 2008; Linck et al., 2014). Therefore, it was deemed to be an essential variable to include in the present study, also given the absence of prior evidence of its role in non-native tone production.

Following on from previous literature, we formulate the following research questions:

**RQ1:** How do L1 tonal status and tone type determine phonetic production accuracy in an imitation task of pseudowords by English-L1 and Mandarin-L2 speakers?
**RQ2:** How do L1 tonal status and tone type determine phono-lexical production accuracy in a picture-naming task of pseudowords by English-L1 and Mandarin-L2 speakers?
**RQ3:** How do an individual’s musical experience, pitch perception aptitude, and WM additionally modulate phonetic and phono-lexical production accuracy?

This study aims to provide a more direct comparison between tonal and non-tonal speakers by observing tone production in a tone system that is unknown to all participants. This enables us to observe more directly how individual language-specific and extralinguistic factors determine tone production in a non-native tone system at the earliest stages of learning. To the best of the authors’ knowledge, there are only two published studies that compared non-native tone production by tonal and non-tonal L1ers (Hao, 2012; A. Yu et al., 2022); however, participants in these studies had previous knowledge of the target L2, which may have affected their production accuracy. Moreover, it appears that the aforementioned cross-linguistic production studies (Hao, 2012; Y. Wang et al., 2003; Yang, 2019; A. Yu et al., 2022) did not strictly control for individual musical experience, pitch aptitude, or WM. The present study investigated the effects of L1 tonal status, tone type, and extralinguistic factors on non-native tone production, while systematically varying or tightly controlling all of the variables concerned.

## 3 Method

### 3.1 Participants

Twenty-one native speakers of English (*f* = 11) and 20 native speakers of Mandarin Chinese (*f* = 10) recruited at the University of Cambridge participated in this study. They also took part in the study reported in Laméris and Post (2022), which examined individual performance in tone perception and word learning in the listening modality. As these data were collected at the same time, they allowed for some cross-modality comparisons of non-native tone processing, as we will touch upon in Section 5. An overview of participants’ demographics is provided in Table 1 and a detailed description is provided in Appendices A and B. None of the English participants had knowledge of a pitch accent or tone language. Within each group, about half of the participants were trained musicians, which meant that they had more than 6 years of formal musical training and that they practiced at the time of the study (Cooper & Wang, 2012; Wong & Perrachione, 2007). WM was operationalized by individual scores obtained in a backwards digit span task. Individual pitch aptitude was operationalized by individual accuracy scores in a tone categorization task. In the tone categorization task, participants heard a rising, a falling, a mid-level, or a low-level tone on a meaningless vowel and were asked to categorize it by selecting an arrow that represented the tone type. For brevity, we refer to the Supplementary Material for a detailed methodology of the digit span and tone categorization tasks, and to Laméris and Post (2022) for a detailed analysis of the tone categorization task results. Equivalence tests (Lakens et al., 2018) with Cohen’s *d* set at 0.5 revealed that the English and Mandarin groups were similar in terms of their measures of musical experience, pitch aptitude, and WM.

**Table 1.** Participant Demographics.

	English (*n* *=* 21)		Mandarin (*n* *=* 20)	
Age (years)	20.98 (1.56)		22.63 (3.32)	
Musical experience (years)	MU (*n* = 11)	NM (*n* = 10)	MU (*n* = 10)	NM (*n* = 10)
	13.32 (2.84)	0.90 (1.66)	14.84 (5.09)	0.98 (0.97)
WM score (%)	57.90 (23.19)		72.09 (23.54)	
Pitch aptitude (%)	89.79 (13.91)		95.22 (4.92)	

*Note.* Values are means with standard deviations in parentheses. MU: musicians; NM: non-musicians; WM: working memory.

### 3.2 Stimuli

**Figure 1.**
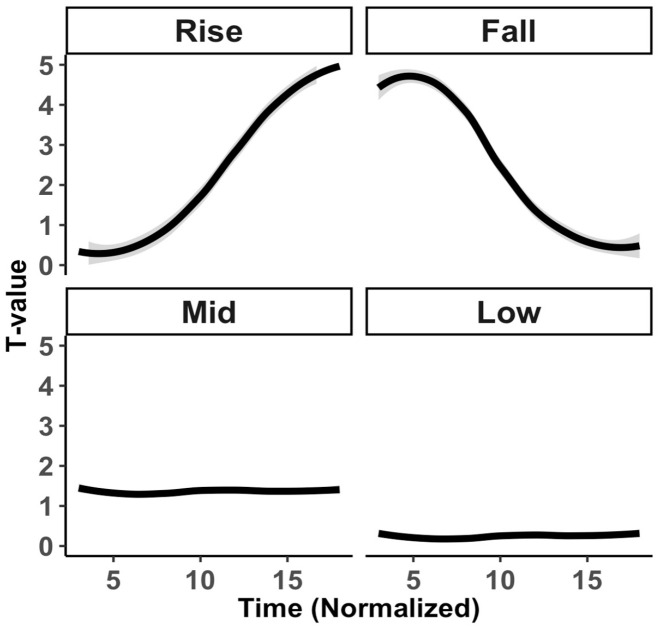
Normalized smoothed pitch tracks of target stimuli per tone. *Note.* Shading ribbons, where present, indicate a 95% confidence interval.

Sixteen pseudowords were used for the imitation and picture-naming tasks. These words were made up of a combination of segments (/nɔn/, /lɔn/, /jɑɹ/ and /juɹ/) and tones (rising, falling, mid-level, and low-level) resulting in 4 × 4 = 16 tone word stimuli. Sound files for the stimuli are in the Supplementary Material. Figure 1 shows the normalized pitch tracks of the target stimuli normalized to *T*-values (Shi et al., 2011). In terms of Chao numerals (Chao, 1968), the four tones can be described as 15 (rise), 51 (fall), 22 (mid-level), and 11 (low-level).

For reference, Mandarin tone categories are typically described as high-level 55, high-rising 35, low-dipping 214, and high-falling 51 (Hao, 2012, p. 270). The four pseudoword tones were chosen explicitly to assess the effect of tone type on English and Mandarin participants. Based on perceptual evidence in terms of the phonetic-acoustic nature of tone types (Francis et al., 2008; Gandour & Harshman, 1978; Qin & Jongman, 2016), the level tone contrasts may be relatively difficult for Mandarin speakers and the contour tones may be relatively difficult for English speakers. In phonological-categorical terms too, the rising and falling tones may be relatively easy for Mandarin speakers because these could assimilate in a one-to-one fashion to the Mandarin rise and fall categories. By contrast, the mid-level and low-level tones may be relatively difficult to process because these could assimilate in a many-to-one fashion to the Mandarin high-level or potentially the low-dipping tone (Qin & Jongman, 2016, p. 334; Zhu et al., 2021, p. 4224). As to the phonological similarity with English intonational tone types, the rising and falling tones resemble canonical rising (low-high) and falling (high-low) intonational tone types in Standard Southern British English (SSBE). The mid-level and low-level tones do not clearly map onto any intonational tone type, as there are no intonational categories in SSBE that are distinguished exclusively by pitch height (Grabe et al., 2003, 2004; Ladd, 2012, p. 91). If we assume that English speakers assimilate non-native tones to intonational types, this too may make the rising and falling tones relatively easy and the level tones relatively difficult. However, given that it remains relatively unclear if tone-to-intonation assimilation occurs in the first place—and even if it does, whether it exerts a strong effect on non-native tone processing (Best, 2019, p. 5; So & Best, 2010; A. Yu et al., 2022, p. 21)—we refrain from making strong predictions about the relative difficulty of the tone types for the English speakers based on their phonological similarity to English intonational types.

Pseudoword stimuli were recorded by a male speaker of Italian, to avoid stimuli recorded by a native speaker of either of the participant group’s languages (Braun & Johnson, 2011; Dupoux et al., 1997). Stimuli were recorded in a sound-attenuated booth at a sampling frequency of 48 kHz. The speaker was instructed to produce stimuli with a flat tone at a comfortable pitch level. The F0 trajectory of this naturally produced flat tone was taken as a baseline tone (the mid-level tone). The speaker was also instructed to naturally produce stimuli with a rising, falling, and low-level tone. Based on the pitch trajectories of these natural productions, the mid-level tone stimuli were then resynthesized using Pitch-Synchronous Overlap and Add (PSOLA) in *Praat* (Boersma & Weenink, 2019) to create stimuli for the other tones. This ensured that tone minimal quadruplets only differed in F0 and not in other acoustic cues while staying as closely as possible to the natural production in terms of the shape of the F0 trajectory. After resynthesis, the average stimulus intensity was set to 70 dB (using the “scale intensity” command in Praat). Five phoneticians deemed the synthesized stimuli to sound as natural as the original mid-level stimuli.

**Figure 2.**
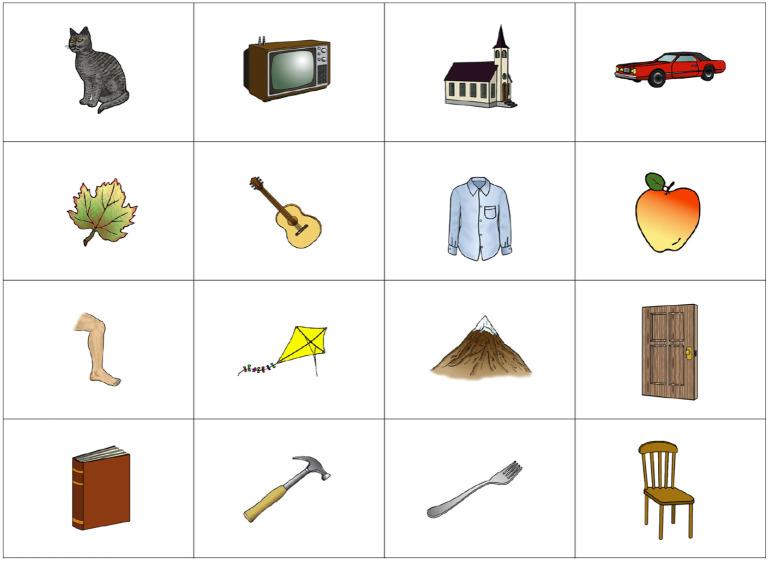
Pictures representing the pseudoword meaning.

Each pseudoword was linked to a picture to establish a sound-meaning connection (Table 2, Figure 2). The pictures were gathered from a database by Rossion and Pourtois (2004) and represent 16 high-frequency nouns (Battig & Montague, 1969; van Overschelde et al., 2004). Care was taken to select lexical items that were semantically unrelated to each other to facilitate word learning (Nation, 2000) and to avoid any obvious phonological similarities between the pseudowords and the corresponding words in English or Mandarin.

**Table 2.** Pseudowords With Corresponding Words in English and Mandarin (in Pinyin Romanization).

Rising	Falling	Mid-level	Low-level
/nɔn15/ *television; diànshì*	/nɔn51/ *book; shū*	/nɔn22/ *cat; māo*	/nɔn11/ *fork; chāzi*
/lɔn15/ *chair; yǐzi*	/lɔn51/ *leg; tuǐ*	/lɔn22/ *apple; píngguǒ*	/lɔn11/ *church; jiàohuì*
/jɑɹ15/ *mountain; shān*	/jɑɹ51/ *kite; fēngzheng*	/jɑɹ22/ *leaf; yè*	/jɑɹ11/ *shirt; chènshān*
/juɹ15/ *door; mén*	/juɹ51/ *guitar; jítā*	/juɹ22/ *car; chē*	/juɹ11/ *hammer; chuízi*

### 3.3 Procedures

A battery of eight tasks was conducted over two consecutive days (Table 3). Note that in addition to the tasks reported in this article (imitation and picture-naming), participants also completed a tone categorization task to measure pitch aptitude, a word identification task to measure lexical tone perception, and a backward digit span task to measure participants’ WM. We refer to Laméris and Post (2022) for a detailed description of these tasks (a description of the tone categorization and backward digit span task is also provided in the Supplementary Material).

**Table 3.** Overview of Tasks.

Description	Duration (min)
Day 1	
Tone categorization (pitch aptitude)^a^	5
Imitation (phonetic accuracy)	10
Picture-naming (phono-lexical accuracy)	5
Word identification^a^	15
Day 2	
Backwards digit span (WM)^a^	5–10
Imitation (phonetic accuracy)	10
Picture-naming (phono-lexical accuracy)	5
Word identification^a^	15

*Note.* WM: working memory.

a Reported in Laméris and Post (2022).

Participants were told that they were taking part in a study that investigated the effects of audiovisual presentation on non-native word learning. After signing a consent form, participants took the tasks individually. The first author only intervened at the start of new tasks to provide instructions. Written instructions for the experiments on the screen were in English or Mandarin depending on the participant’s native language. The experiment was carried out over 2 days to limit the total time spent in one session and to facilitate word recall after a night of sleep (Dumay & Gaskell, 2007).

All experiments were administered in a sound-attenuated booth and run on a *DELL Inspiron 13 5000 Series* touchscreen tablet laptop through the *OpenSesame* software (Mathôt et al., 2012). Participants listened to audio stimuli over *Beyerdynamic DT 990* headphones at a comfortable listening level. Voice recordings were made using a *Sennheiser* cardioid microphone and *SoundDevices mixpre6* recorder at a sampling frequency of 48 kHz.

#### 3.3.1 Imitation task

The imitation task served to measure phonetic tone production accuracy and to train participants to memorize novel L2 words for the subsequent picture-naming task in a relatively efficient way through mimicry (Baills et al., 2019; Li & Dekeyser, 2017). Prior to the imitation task, there was a familiarization phase in which participants saw each picture, accompanied by the picture’s meaning written in English or Mandarin, and named the picture in their native language. This ensured that participants considered the pictures to be analogous to a word in their L1.

After the familiarization phase, participants started the imitation task. Participants were instructed to listen carefully to each pseudolanguage word and to repeat it out loud as accurately as possible immediately after the word was presented while simultaneously trying to memorize its meaning. Productions were voice-recorded and participants received no feedback regarding their production. Each of the 16 pseudowords was audiovisually presented four times, resulting in 64 trials in total. Participants had 5000 ms to repeat the word before the next audiovisual stimulus was presented. The first two trials were in a pseudorandomized order for all participants: each audiovisual stimulus was presented twice in a row (e.g., the word for “cat,” followed by the participant’s imitation, followed by one more trial [presentation + imitation] for “cat”). The order was such that no segmental or tonal minimal pair followed one another. The last two presentations were fully randomized for each participant individually.

The same imitation task was conducted on Day 2. The only difference was that the picture familiarization was not conducted, and that the pseudorandomized presentation order was the reverse of that of Day 1.

Although the nature of an imitation task is inherently pre-lexical, as it requires a participant to listen to an auditory stimulus and accurately reproduce it immediately after, which does not necessitate lexical retrieval, it is not unthinkable that our imitation task also involved some lexical processing. This is because upon hearing the auditory stimulus, participants also saw the picture that represented the meaning of a word, and were gradually establishing a sound-meaning connection through the course of the experiment. We will discuss the possible effects that any top-down processing from the lexical level on imitation performance may have had in Section 5. However, we still deemed the imitation task to be a suitable method to investigate differences in tone production in terms of *phonetic accuracy*, given that participants were all expected to reliably reproduce tones according to their broad phonological properties, and only show differences in fine-grained phonetic properties.

#### 3.3.2 Picture-naming task

The picture-naming task, which replicates L1-to-L2 recall abilities (Barcroft & Sommers, 2014; Li & Dekeyser, 2017), served to measure phono-lexical accuracy of tone production. The picture-naming task was held directly after the imitation task. Each of the 16 visual stimuli was presented one by one, in a randomized order, for 5,000 ms through the software *OpenSesame*. Participants were asked to pronounce the pseudolanguage word to the best extent of their memory. Their productions were voice-recorded. The exact same task was repeated on Day 2.

### 3.4 Determining tone production accuracy

#### 3.4.1 Imitation task: phonetic production accuracy

Because of a recording error on Day 1, only the imitations from the first block (in which each word was imitated twice) were analyzed. The target stimuli productions and participants’ imitations were first segmented in *Praat*, after which F0 values were extracted with *ProsodyPro* (Xu, 2013) at 20 equidistant points for each pseudoword. Because of a relatively high proportion of pitch excursions at the edges of the measurements, the first and last two measurements from the obtained trajectories were truncated. Any other pitch excursions or missing values within the remaining 16-point trajectories were manually corrected by interpolation or by re-obtaining the F0 values using the *Pitch Listing* command in *Praat*. Twenty-one out of 2,264 participant data points (2 imitations × 16 words × 41 participants × 2 days) were excluded due to recording errors or non-responses, resulting in a final total of 2603 data points for analysis. F0 values were normalized to T-values (Shi et al., 2011), which are similar to Chao values, to account for individual differences in pitch range before any further analysis, using Equation 1:

(1) $$F_{0}=5\times \frac{\log_{10}x_{i}-\log_{10}x_{min}}{\log_{10}x_{max}-\log_{10}x_{min}}$$

To determine phonetic tone production accuracy in the imitation task, we conducted DCT analyses of the normalized F0 trajectories, following the same methodology described in A. Yu et al. (2022). DCT reduces the F0 trajectories into a given number of coefficients (in this case three: DCT1, DCT2, and DCT3). These three coefficients are proportional to an F0 trajectory’s mean pitch height, its slope, and its curvature, respectively. We obtained DCT coefficients for the target stimuli and for the individual imitations by the English and Mandarin participations. This enabled us to investigate whether individual imitations were similar (or dissimilar) to the target stimuli in terms of pitch height, slope, and curvature.

Furthermore, and following A. Yu et al. (2022), we obtained the tonal distance from the imitations to the tonal centroid of the target stimuli to capture the overall relative distance from individual imitations to the target. Taking the normalized duration and DCT coefficients of the target stimuli as the centroids, we calculated the Euclidean distance from each tone production of each speaker to the respective tonal centroid. The code for the tonal distance and DCT analyses can be found in the Supplementary Material.

We will consider the tonal distance as a main proxy for phonetic accuracy. In this way, a larger tonal distance indicates a less target-like, and thus, less phonetically accurate imitation. In other words, the tonal distance allows us to investigate *whether* an imitation was phonetically (in)accurate, and the DCT coefficients further tell us *how* an imitation was phonetically (in)accurate in terms of pitch height, slope, and curvature.

#### 3.4.2 Picture-naming task: phono-lexical production accuracy

The picture-naming task served to measure phono-lexical accuracy. Recall that phono-lexical accuracy in this article is defined by the ability to produce overt phonological contrasts (segmental and tonal) to distinguish word meaning, for instance, in contrasting /a/ and /u/ and a rising and a falling tone to respectively distinguish “mountain” /jaɹ15/ from “guitar” /juɹ51/. To this end, phono-lexical accuracy was calculated based on manual labeling by the first and second authors (the second author was not involved with the present study at the time of labeling). Labels for segments and tones were assigned based on auditory observations and inspection of formants and F0 trajectories in *Praat.* Inter-rater reliability was determined using the *irr* package (Gamer et al., 2019) in *R 4.1.1* (R Core Team, 2021). *Kappa* statistics were .96 for segmental and .86 for tonal labels, indicating a very strong level of agreement. In case of disagreement, the label suggested by the second rater was applied.

Although it was relatively unequivocal to determine whether a production in the picture-naming task should be labeled “rise” or “fall” based on an auditory impression and on F0 trajectory observations, it was sometimes less clear whether a production should be labeled as “mid-level” or “low-level.” To disambiguate these tone productions, the *Contour Clustering* Graphical User Interface (Kaland, 2021) was used to determine in a more objective way whether participants produced distinct mid-level and low-level tone categories. The *Contour Clustering* GUI carries out an automated data-driven cluster analysis that groups together F0 contours based on their acoustic similarities into a defined number of clusters (see Kaland, 2021 for details).

F0 trajectories of picture-naming productions (per participant and per day) that were labeled as level tones were imported in the *Contour Clustering* GUI and reduced to two clusters to determine whether participants made a categorical distinction between mid-level and low-level tones. The results revealed that 91.22% of the labels that were initially assigned by the raters corresponded to mid-level and low-level clusters generated by the GUI. The 52 non-corresponding labels (e.g., a word that was initially labeled as a mid-level tone by the raters but that was clustered in the low-level category by the GUI) were changed accordingly in line with the categorization proposed by the *Contour Clustering* GUI.

Finally, there were a few instances (2.80% of all productions) in which segmental productions were clearly deviant from the target words, such as /weɹ/ or /na/. These productions were labeled as incorrect non-target-like responses. There were only two instances in which tone productions were not clearly categorizable into any of the four categories, and these were removed from the analysis. Four out of 1,312 data points (16 productions × 41 participants × 2 days) were removed from analysis due to bad recording quality or non-responses, resulting in a final total of 1,308 data points.

### 3.5 Statistical procedures

We used Bayesian inference to investigate the effects on L1 tonal status, tone type, and extralinguistic factors on phonetic accuracy in imitation and phono-lexical accuracy in picture-naming. Models were fitted using the *brms* package (Bürkner, 2018) in *R 4.1.1* (R Core Team, 2021). We use terminology based on Nicenboim et al. (2018, p. 1079) to interpret parameter estimates of the posterior distribution of a Bayesian model. We assume that there is “suggestion” for an effect if zero lies outside the 95% credible interval (CrI). We assume “weak suggestion” for an effect if zero is included in the 95% CrI but the maximum probability of direction (*pd*) is relatively high. Finally, we assume that there is no suggestion for an effect if the maximum probability of direction is near 50%.^2^

Following common practice (Haendler et al., 2020; Vasishth et al., 2018), we constructed models with (very) weakly informative priors with the mean centered around zero and a standard deviation of 10 for all population- and group-level regression coefficients and LKJ(2) for correlation priors. Four sampling chains with 3000 iterations each were run with 1500 warm-up iterations. Models for phonetic accuracy in imitation (dependent variable = tonal distance, DCT score) were fitted with a Gaussian distribution. The models for phono-lexical accuracy in picture-naming (dependent variable = correct/incorrect) were fitted with a Bernoulli distribution. We also fitted a model on the count of error types in picture-naming, which was fitted with a zero-inflated Poisson distribution, as is suitable for count models with an excess number of zeroes (Winter & Bürkner, 2021). Model diagnosis was carried out by observing Rhat values (ensuring these were close to 1), and by inspecting posterior draws using the pp_draws() command of the *brms* package.

We fitted models including fixed effects and interactions of interest. An overview of each model is provided in Table 4. All categorical fixed effects were contrast-coded, and continuous fixed effects were centered and scaled.

**Table 4.** Overview of Models.

Task	Model name	Dependent variable
Imitation	Tonal distance^a^	Tonal distance from target per imitation
	DCT^b^	DCT coefficient per imitation
Picture-naming	Picture-naming^c^	Correct/incorrect per trial
	Error type count^d^	Count per tone-only error type

*Note.* The model formulae (brm) are as follows:

a Distance ~ L1 * tone * day * (ME + WM + PA) + (1 + tone + day|subject) + (1|item).

b DCT ~ group * tone * day + (1 + tone + day|subject) + (1|item).

c Correct ~ L1 * tone*(ME + WM + PA) + (1 + tone|subject) + (1|item).

d Count ~ L1 * error_type + (1 + error_type|subject).

DCT: discrete cosine transformation; ME: musical experience; WM: working memory; PA: pitch aptitude.

For the imitation task, the model for the tonal distance between participants’ and target imitations contained categorical fixed effects for *L1* (English, Mandarin), *Tone Type* (Rise, Fall, Mid-level, Low-level), *Day* (Day 1, Day 2),^3^ and continuous fixed effects for *Musical Experience* (years of formal practice), *Pitch Aptitude* (tone categorization accuracy), and *Working Memory* (WM score). The model contained four-way and lower-level interactions between the *L1*, *Tone Type*, *Day*, and each of the continuous variables.

The model predicting DCT coefficients contained categorical fixed effects of *Group* (Target, English, Mandarin), *Tone Type*, and *Day* and three-way and lower-level interactions between these variables.

Both models contained by-subject random slopes for *Tone Type* and *Day*, and a random intercept for *Item*.

The model for correct picture-naming likelihood contained fixed effects for *L1, Tone Type, Musical Experience, Pitch Aptitude*, and *Working Memory*. As will be explained in Section 4, we only report results from Day 2. The model contained three-way and lower-level interactions between the categorical and each of the continuous fixed effects, a by-subject random slope for *Tone Type*, and a random intercept for *Item*.

The model that predicted the count of error types in the picture-naming task contained fixed effects of *L1* and *Error Type* and two-way interaction between *L1* and *Error Type*, and a by-subject random slope for *Error Type*.

When there was a suggestion for an interaction effect (i.e., zero not included in the 95% CrI), we carried out multiple comparisons with the *emmeans* package (Lenth, 2020). We assume that multiple comparisons are meaningful when zero is not included in the 95% highest posterior density (HPD) as calculated by the *emmeans* package.

For brevity, the complete statistical results (posterior distributions and multiple comparison tables) are reported in the Supplementary Material. In Section 4, we highlight findings for which we found suggestion for an effect (i.e., zero not included in 95% CrIs or HPDs).

### 3.6 Predictions

The following predictions are formulated with regard to our research questions:

**RQ1:** Based on the reviewed literature, we predict that any differences between English-L1 and Mandarin-L1 participants in phonetic accuracy in the imitation task will be due to tone type rather than L1 tonal status. Specifically, we predict that English participants may be better than Mandarin participants in the accurate imitation of mid-level and low-level tones, but worse than Mandarin participants in the accurate imitation of rising and falling tones.
**RQ2:** We predict that L1 tonal status will interact with tone type in similar ways as in the imitation task to determine phono-lexical accuracy in the picture-naming task. However, there is also a possibility that Mandarin speakers will outperform English speakers overall in the picture-naming task, given that tonal L1ers may be better than non-tonal L1ers to link tonal representations to lexical meaning, cf. Poltrock et al. (2018).
**RQ3:** Finally, we predict a facilitative effect of musical experience, pitch aptitude, and WM on imitation and picture-naming performance.

## 4 Results

### 4.1 Imitation: phonetic accuracy

#### 4.1.1 Visualization of imitations

**Figure 3.**
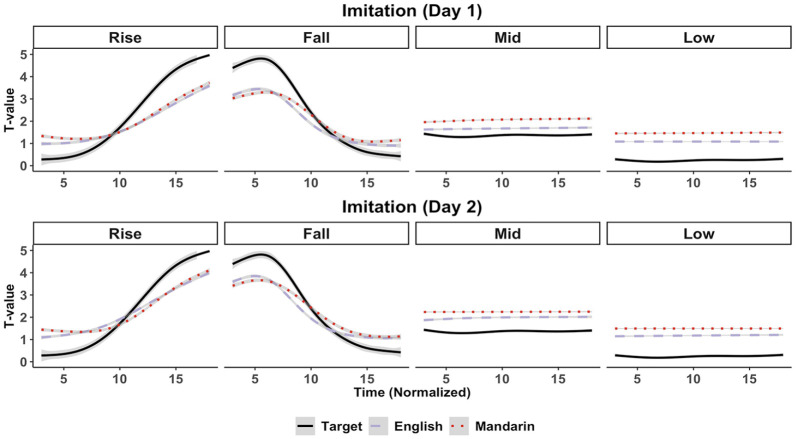
Imitation: group-averaged pitch trajectories. *Note.* Shading ribbons, where present, indicate a 95% confidence interval.

Figure 3 shows group-averaged pitch trajectories of the imitations by the English and Mandarin participants. The target stimuli trajectories are shown as reference. A visual inspection of the English and Mandarin imitations reveals two main deviations from the target. First, pitch range for the rising and falling tones is relatively compressed. At least on Day 1, it appears that participants do not attain high pitch values at the extremes of these contour tones. Second, productions for the mid-level and low-level tones appear to be relatively high in comparison to the target values.

#### 4.1.2 Tonal distance

**Figure 4.**
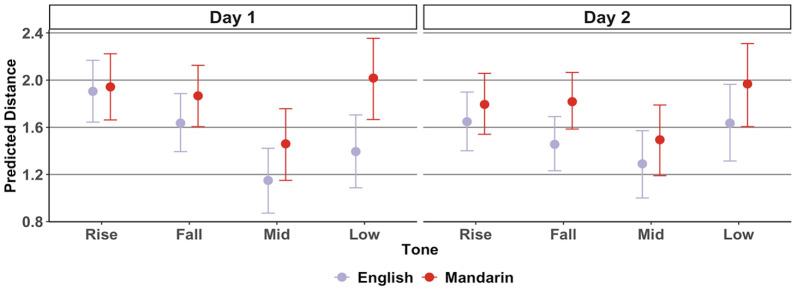
Imitation: predicted mean tonal distance per L1, tone, and day. *Note.* Bars represent a 95% credible interval.

The predicted tonal distance is shown in Figure 4. A visual inspection suggests that on Day 1, both English and Mandarin imitations had relatively large tonal distances from the target (i.e., relatively low phonetic accuracy) for rising and falling tones. It also appears that imitations of low-level tones had relatively large tonal distances from the target for the Mandarin group. A similar pattern can be observed on Day 2, although the distances seem to become shorter.

The tonal distance model revealed an *L1:Tone:Day* interaction, *b* = 0.04 (0.01, 0.08). Multiple comparisons suggested that on Day 1, Mandarin speakers’ low-level tone imitations had a larger distance than that of English speakers, *b* = 0.62 (0.24, 1.02). On Day 2, Mandarin speakers’ falling tone imitations had a larger distance than that of English speakers, *b* = 0.36 (0.14, 0.59).

Multiple comparisons across days further suggested that tonal distance decreased over the 2 days for English speakers for rising *b* = −0.25 (−0.39, −0.12) and falling tones, *b* = −0.18 (−0.30, −0.03), but that distance increased for mid-level, *b* = 0.14 (0.01, 0.27) and low-level tones, *b* = 0.24 (0.10, 0.37). There was a weak suggestion that tonal distance decreased for Mandarin imitations of rising tones, with zero included in the 95% HPD, *b* = −0.15 (−0.31, 0.00).

The model also revealed a *Tone:Day:Musical Experience* interaction, *b* = −0.04 (−0.09, 0.00), suggesting that the effect of musical experience differed per tone and per day. Multiple comparisons of the effect of musical experience per tone and per day revealed small differences in the effect size of musical experience on tonal distance, but there was no suggestion that musical experience affected tonal distance in any condition, with all HPDs containing zero. There was no suggestion for an overall effect of musical experience on tonal distance, *b* = −0.03 (−0.16, 0.09), *pd* = 69.72%.

The model revealed an *L1:Tone:Day:Pitch Aptitude* interaction, *b* = 0.10 (0.05, 0.16). Multiple comparisons of the effect of pitch aptitude in each sub-condition (per L1, day, and tone) suggested that pitch aptitude was associated with smaller tonal distance for Mandarin participants’ imitations of falling tones on Day 2, *b* = −0.59 (−0.94, −0.28) but with larger tonal distance for Mandarin imitations of mid-level tones on Day 2, *b* = 0.67 (0.18, 1.13).

There was only a weak suggestion for an effect of *WM*, *b* = −0.09 (−0.19, 0.02), *pd* = 94.37%. The negative estimate would suggest that higher individual WM score was associated with smaller tonal distance.

#### 4.1.3 DCT analysis

**Figure 5.**
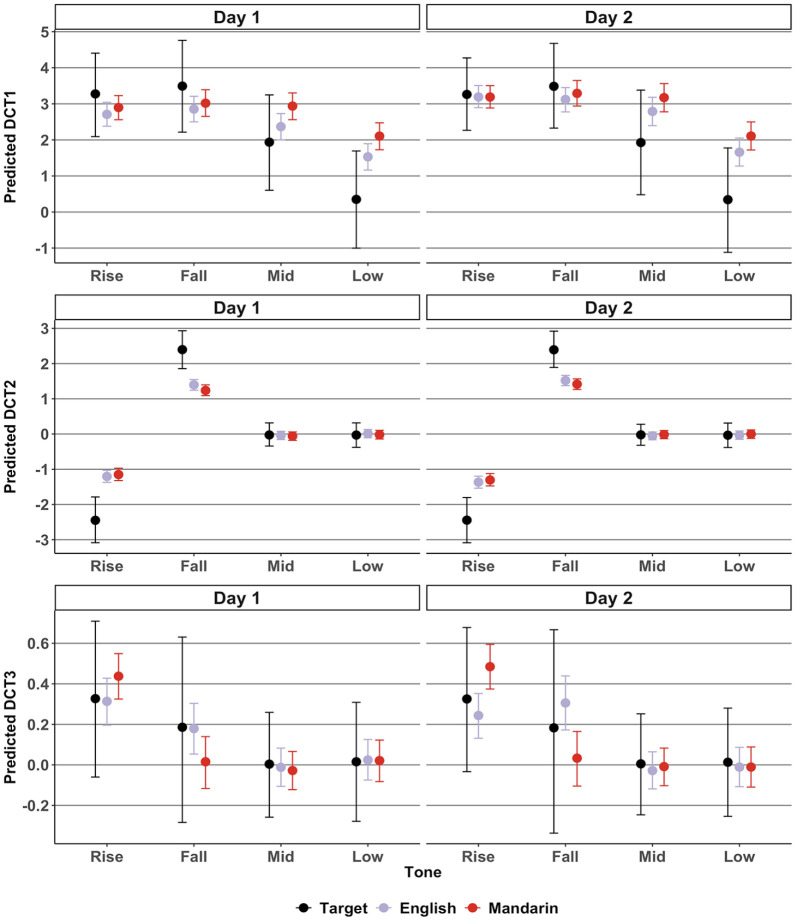
Imitation: predicted mean DCT coefficients per group, tone, and day. *Note.* Bars represent a 95% credible interval.

In this section, we present results from the DCT analyses. Unlike the tonal distance, which revealed *whether* an imitation was target-like or not, the DCT coefficients further reveal *how* the imitations were target-like in terms of relative mean pitch height (DCT1), slope (DCT2), and curvature (DCT3). Figure 5 shows the predicted three DCT coefficients of the target tones and of the imitations by the English and Mandarin groups.

DCT1 correlates with mean pitch height. A larger DCT1 coefficient indicates a higher mean pitch. A visual inspection of Figure 5 shows that DCT1 coefficients are relatively large for participants’ imitations of the mid and low-level tones compared with the target, suggesting that imitations were relatively high in pitch. This reflects the observation of the pitch trajectories in Figure 3. The DCT1 model suggested a *Group:Tone* interaction, *b* = 0.46 (0.03, 0.88). Multiple comparisons revealed that, averaged over the 2 days, DCT1 coefficients for mid-level tones were larger in the Mandarin group compared with the English group, *b* = 0.47 (0.08, 0.87). In addition, DCT1 coefficients for low-level tones were larger for the Mandarin group compared with the English group, *b* = 0.51 (0.13, 0.92) and the target, *b* = 1.75 (0.36, 3.01). This suggests that participants’ imitations of level tones were relatively high in pitch, and particularly so for Mandarin speakers’ low-level tone imitations, which were higher in mean pitch compared with both the target and the English group.

DCT2 correlates *negatively* with slope. This can be observed in the coefficients in Figure 5. Rising tones having a negative DCT2, and falling tones have a positive DCT2. Accordingly, DCT2 for the mid-level and low-level tones’ DCT2 is virtually zero. The DCT2 model suggested a *Group:Tone*, *b* = −0.76 (−1.08, −0.42) and a *Tone:Day*, *b* = 0.05 (0.01, 0.09) interaction. Multiple comparisons revealed that, averaged over the 2 days, DCT2 coefficients for target rising tones were smaller in comparison to both the English, *b* = −1.16 (−1.78, −0.54) and the Mandarin groups, *b* = −1.22 (−1.82, −0.57). This suggests a *more positive* slope for the target rising tone compared with the English and Mandarin imitations. DCT2 coefficients of the target falling tones were larger in comparison with both the English, *b* = 0.94 (0.43, 1.45) and the Mandarin groups, *b* = 1.07 (0.55, 1.56). This suggests a *more negative* slope for the target falling tone compared with the English and Mandarin imitations. In other words, the slopes of English and Mandarin rising and falling tone imitations were less steep than the target slopes.

Despite the suggestion for a *Tone:Day* interaction, multiple comparisons between *Tone* and *Day* revealed no clear differences in DCT2 between tones across days or vice-versa. Therefore, to better understand what may have been behind the *Tone:Day* interaction, multiple comparisons for DCT2 between Day 2 and Day 1 per *Group* and per *Tone* were conducted exploratorily. Given that there was only a weak suggestion for a three-way *Group:Tone:Day* interaction, *b* = −0.05 (−0.13, 0.02), *pd* = 91.80%, these comparisons must be interpreted with caution. It suffices to say here that these comparisons suggested that DCT2 decreased for rising tones from Day 1 onto Day 2 for both English and Mandarin groups, and that it increased for falling tones from Day 1 onto Day 2 for both groups, with the estimates and 95% HPDs falling completely above or below zero. For the target stimuli, there was (obviously) no difference in DCT2 across days, with the estimated mean difference approaching zero. Overall, this may suggest that the slopes of participants’ imitations became steeper on Day 2 of the imitation task.

DCT3 correlates positively with curvature. A visual inspection of Figure 5 reveals relatively high DCT3 coefficients for the contour tones (rising and falling) and coefficients of nearly zero for the level tones (mid-level and low-level). The model for DCT3 suggested a *Group:Tone:Day* interaction, *b* = −0.04 (−0.08, −0.01). Multiple comparisons revealed that DCT3 was smaller for English rising tone imitations compared with Mandarin speakers’ imitations on Day 1, *b* = −0.12 (−0.24, −0.01) and Day 2, *b* = −0.24 (−0.35, −0.13). However, DCT3 was larger for English falling tone imitations compared with Mandarin speakers’ imitations on Day 1, *b* = 0.16 (0.03, 0.31) and Day 2, *b* = 0.27 (0.12, 0.42). Finally, the comparisons revealed that, from Day 1 to Day 2, DCT3 increased for English falling tones, *b* = 0.13 (0.06, 0.18) but decreased for English rising tones, *b* = −0.07 (−0.13, −0.01).

### 4.2 Picture-naming: phono-lexical accuracy

Overall accuracy in the picture-naming task is shown in Table 5. As is shown by the accuracy scores (69% for English and 71% for Mandarin participants), participants on average were able to correctly name most of the pictures by producing the pseudoword correctly in both segmental and tonal properties by the end of the word training session on Day 2.

**Table 5.** Picture-Naming: Descriptive Statistics.

	English	Mandarin
Day 1 % correctly named	29.5 (20.8)	30.7 (14.7)
Day 1 % of tone-only errors	26.7 (14.2)	35.8 (15.5)
Day 2 % correctly named	68.6 (29.4)	70.9 (20.3)
Day 2 % of tone-only errors	53.2 (30.3)	49.8 (33.1)

*Note.* Values are means with standard deviations in parentheses.

Because this study concerns phono-lexical *tone* accuracy, which reflects the ability to link tonal and segmental categories to a lexical representation, and not a general ability to associate sounds with meaning, the following analyses will focus solely on tone accuracy and “tone-only errors” (Wong & Perrachione, 2007). A tone-only error is an error in which a participant incorrectly names a picture based purely on its tonal properties. For instance, a tone-only error would be an error in which a participant incorrectly names the picture for “television” as /nɔn22/ instead of the target /nɔn15/. Other types of picture-naming errors, for instance, incorrectly naming the picture for television /nɔn22/ as /jaɹ11/, are not directly indicative of a participant’s inability to associate tone to lexical meaning. Such errors may instead indicate that a participant had simply not memorized the overall phono-lexical information. Therefore, to focus on *tone* word learning facility in the present analysis, we report picture-naming results of items for which participants named at least the segmental properties correctly. Furthermore, we only present data from Day 2. This is because on Day 1, the accuracy scores (ca. 30%) and the proportion of tone-only errors were relatively low. Tone-only errors only constituted 26.67% (*SD* = 14.18) of English participants’ and 35.81% (*SD* = 15.49) of Mandarin participants’ total number of errors on Day 1 (as shown in Table 5). This suggests that participants were still learning the overall sound-meaning connection and had not yet reached a stage at which they could start focusing on accurately producing both the segmental and tonal properties. On Day 2, the mean proportion of tone-only errors increased to 53.21% (*SD* = 30.30) and 49.82% (*SD* = 33.13) for the English and Mandarin groups, respectively.

In line with our research questions, we first report how correct picture-naming likelihood differed per L1 and per tone type. We then report how extralinguistic factors (musical experience, WM, and pitch aptitude) further modulated performance.

#### 4.2.1 Effect of L1 tonal status and tone type on phono-lexical tone accuracy

**Figure 6.**
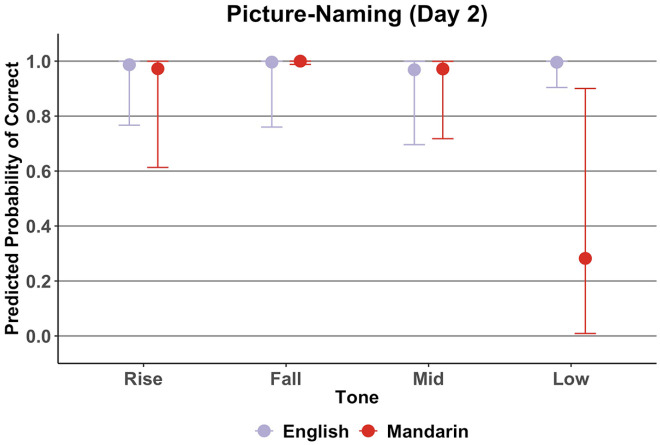
Picture-naming: predicted probability of correct picture-naming per L1 and tone. *Note.* Bars represent a 95% credible interval.

Figure 6 shows the predicted probability of correct picture-naming per L1 and tone type. A visual inspection suggests that Mandarin participants were very likely to correctly name words with falling tones, but less likely to correctly name words with low-level tones, compared with the English participants.

The picture-naming model revealed a weak suggestion for an *L1: Tone* interaction, with zero included in the 95% CrI, although the probability of direction was relatively high, *b* = −2.46 (−5.64, 0.28), *pd* = 97.17%. Multiple comparisons suggested that English speakers were more likely than Mandarin speakers to correctly name low-level tone words, *b* = 6.42 (2.24, 12.85), although this should be interpreted with caution given the weak suggestion for a *L1:Tone* interaction.

#### 4.2.2 Tone-only error types

**Figure 7.**
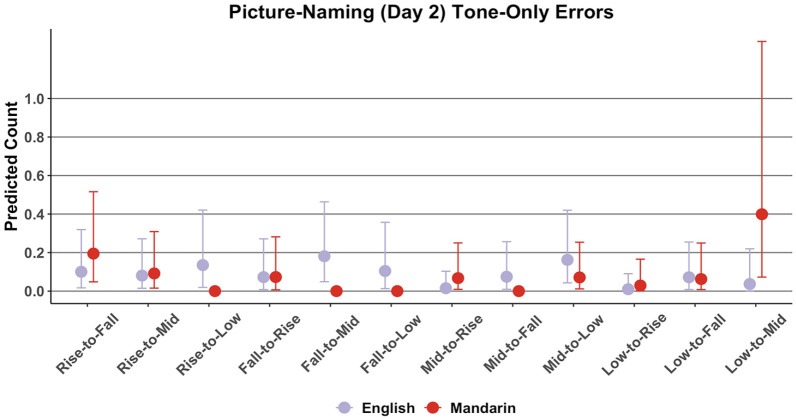
Picture-naming: predicted average counts of tone-only error types. *Note.* Bars represent a 95% credible interval.

To further investigate the nature of participants’ performance per tone, this section reports the count of specific tone-only error types. Figure 7 shows the distribution of predicted average count of errors per tone-only error type. For instance, a “Rise-to-Fall” tone-only error is an error in which a participant incorrectly produced a target rising tone as a falling tone, for example, mispronouncing /nɔn15/ as /nɔn51/. A visual inspection suggests that English participants incorrectly named words in terms of their tonal properties across the board, whereas it appears that Mandarin participants predominantly incorrectly named low-level words as mid-level words.

The error type count model revealed an *L1:Error Type* interaction, *b* = −2.51 (−4.40, −0.96). Multiple comparisons suggested that English speakers made more rise-to-low, *b* = 12.98 (0.85, 33.52), fall-to-mid, *b* = 13.36 (1.17, 33.01), fall-to-low, *b* = 12.86 (0.54, 32.47), and mid-to-fall errors, *b* = 12.43 (0.55, 32.87) than did Mandarin speakers. The comparisons further suggested that Mandarin speakers made more low-to-mid errors than did English speakers, *b* = 2.35 (0.31, 5.39).

#### 4.2.3 Effects of extralinguistic factors

The picture-naming model suggested that picture-naming was facilitated by musical experience, *b* = 2.07 (0.27, 4.21) and pitch aptitude, *b* = 2.63, (0.30, 5.03). There was a weak suggestion for a facilitative effect of WM, *b* = 0.87 (−0.65, 2.51), *pd* = 88.48%.

## 5 Discussion

### 5.1 Effects of L1 tonal status and tone type on phonetic accuracy of non-native tone production

In RQ1, we asked to what extent L1 tonal status and tone type determine individual differences in phonetic accuracy of tone production. We found that phonetic accuracy of non-native tone productions—operationalized by the tonal distance between individual productions and the target stimuli in the imitation task—was not predicted by L1 tonal status in and of itself. Contrary to the intuition that L1 experience with the phonological use of a specific feature could facilitate non-native processing of that feature (McAllister et al., 2002), Mandarin (tonal) L1ers were not more accurate than English (non-tonal) L1ers in their phonetic imitation of non-native pseudowords. However, an observation of performance per tone type revealed L1-specific patterns of phonetic accuracy. Specifically, on Day 1, tonal distance to the target was larger for Mandarin speakers’ imitations of low-level tones in comparison to English speakers. On Day 2, tonal distance to the target was larger for Mandarin speakers’ imitations of falling tones in comparison to English speakers.

Whereas the tonal distance revealed *whether* an imitation was phonetically accurate, the DCT coefficients further revealed *why* an imitation was phonetically accurate. In the case of the low-level tone imitations, the DCT1 coefficients for the Mandarin group were higher than both the target and the English group. This suggests that Mandarin speakers imitated low-level tones with a relatively high pitch. This falls in line with the prediction made for RQ1, namely, Mandarin speakers, who are known to struggle phonetically with pitch height distinctions relatively more than English speakers, and who in addition may have phonologically assimilated the present study’s level tones to their single L1 high-level tone category, may have had a particular difficulty in accurately imitating low-level tones. The observation that low-level tones in particular, and not both mid-level and low-level tones were produced less accurately, may chime in with the notion of a “phonetic residual” (Chen et al., 2020). That is, even when tonal speakers assimilate non-native contrasts to a single L1 tone category in a phonological-categorical way, they may still be sensitive to the tones’ phonetic properties. Our study’s low-level tone (11) is phonetically more deviant from the Mandarin high-level tone (55) than the mid-level (22) tone. This difference could explain why low-level tones were particularly difficult to imitate accurately for Mandarin speakers, cf. Zhang and Peng (2017). We note, however, that any claims regarding assimilation between native and non-native tones can only be “speculative in nature” (Francis et al., 2008, p. 284). This is especially the case in the present study because Mandarin participants were not asked to rate the similarity between their native tones and the non-native tones, as has been done in some previous studies (Chen et al., 2020; Reid et al., 2015).

The observation that Mandarin imitations of falling tones were phonetically less accurate than English imitations (on Day 2) does not fit the prediction for RQ1 that Mandarin speakers should have less difficulty with the production of contour tones compared with English speakers. The DCT analyses revealed that only DCT3 coefficients (which correlate with curvature) differed between Mandarin and English speakers for falling tones. More specifically, the Mandarin DCT3 coefficients were found to be lower (and hence less target-like) than the English DCT3 coefficients. It is puzzling as to why Mandarin imitations would be less “curvy” than English imitations. One possibility is that Mandarin speakers relied on the overall shape of their own falling tone category to imitate the falling tones. It could be said that Mandarin falling tones differ from the pseudoword tones because the Mandarin falling tone can be described as a “straight falling line” (Shih & Lu, 2015) which is realized by means of a drastic fall in pitch over a relatively short duration (Hao, 2012, p. 272; Tupper et al., 2020). It is possible that this difference between the target falling tones and the Mandarin falling tones led to relatively less accurate imitations. In contrast, English speakers may have relied more on the direct acoustic signal obtained from the target stimuli to accurately imitate the falling tone, but this requires more investigation. We also observed that for English speakers, DCT3 increased for falling tone imitations, but decreased for rising tone imitations from Day 1 to Day 2. It is difficult to find a reasonable interpretation for this.

There were also global patterns in terms of phonetic accuracy per tone. For instance, the analysis of DCT2 coefficients suggested that both English and Mandarin speakers differed from the target in terms of the slope of rising and falling tones. A visual inspection in Figure 3 suggests that participants did not fully exploit the tonal space to produce rises and falls with steep slopes. The fact that this was observed in both groups to similar degrees may indicate a general effect of operating in a non-native system, in which speakers may not fully exploit the available pitch range (Grazia Busà & Urbani, 2011; Zimmerer et al., 2014).

Finally, a comparison between phonetic accuracy on Days 1 and 2 allowed us to assess whether phonetic accuracy improved over time. In the English group, tonal distance decreased (i.e., phonetic accuracy increased) from Day 1 onto Day 2 for rising and falling tones. For Mandarin speakers, there was only a weak suggestion that rising tones became more target-like on Day 2. Unexpectedly, tonal distance increased for English speakers’ mid-level and low-level tones, which suggests that imitations became less target-like.

Baese-Berk (2019) proposes two routes along which speakers may improve in imitation. The first is an acoustic-phonetic route, which assumes that speakers improve their imitations by more accurately matching their productions to the target tokens. In the present study, it is plausible that, as a function of increased practice, participants had simply become better at fine-grained phonetic imitation of rising tones, which involves precise control over F0 height, the timing of a change in F0, and the velocity with which this F0 change takes place. Indeed, the DCT2 analysis suggested that both participant groups became more target-like in terms of the slope of rising tones.

The second possible route of improvement in imitation is a phonological one, which assumes that speakers acquire new categories from which they can select exemplars to use in production (Baese-Berk, 2019, p. 998). This scenario could explain the (perhaps surprising) finding that English imitations of level tones were in fact less accurate on Day 2 than on Day 1. It may have been the case that on Day 2 of the imitation task, English participants started relying less on the acoustic signal that they heard, and instead started approximating their imitations to their (perhaps still unstable) phonological representations of pseudoword level tones in a top-down manner, cf. Bock and Levelt (1994). It is unclear why phonetic accuracy for level tones did not decrease in the same way for the Mandarin speakers. One possibility is that phonetic accuracy for level tones was already relatively low on Day 1 (as evidenced by the relatively high DCT1s and tonal distances as observed in Figures 4 and 5), and that this did not change much on Day 2 because Mandarin speakers may have relied on native tone categories throughout.

It should be mentioned that the most important differences observed between English and Mandarin speakers in terms of phonetic accuracy—namely that English imitations had more target-like low-level tones on Day 1, and more target-like falling tones on Day 2—were relatively marginal when looking at overall imitation performance. Indeed, although Mandarin speakers appeared to imitate low-level tones with a higher pitch than did English speakers, this difference in relative pitch height was relatively subtle. English speakers too tended to imitate the target level tones with a relatively high pitch (as observed in Figure 3). In addition, the difference in curvature of the falling tones (DCT3) between English and Mandarin speakers on Day 2 appeared to be relatively small, and there were no differences in terms of the slope (DCT2) or relative pitch height (DCT1) of the falling tones between the two groups. Thus, although we observed these subtle differences in phonetic accuracy, which may have been due to an interaction between L1 and tone type, overall performance in the imitation task was relatively uniform between the two groups.

### 5.2 Effects of L1 tonal status and tone type on phono-lexical accuracy of non-native tone production

For RQ2, we predicted that L1 tonal status and tone type would jointly determine phono-lexical accuracy in the picture-naming task, although we considered the possibility that Mandarin speakers might outperform English speakers given their familiarity with linking tonal categories to lexical meaning. Our results showed that Mandarin participants’ L1 tonal status did not facilitate phono-lexical accuracy. Mandarin participants did not outperform English participants in the picture-naming task, nor did they produce fewer tone-only errors than did English participants. This finding suggests that, at the very first stages of encountering a novel tone system, linking tones to lexical meaning in the speaking modality may not necessarily be easier for tonal L1ers than for non-tonal L1ers.

However, there were L1-specific patterns in picture-naming performance per tone type. Most notably, there was a weak suggestion that Mandarin speakers were less likely than English speakers to correctly name pictures that represented pseudowords with low-level tones. An analysis of the type of tone-only errors types further revealed that Mandarin speakers predominantly mispronounced low-level tone words as mid-level tone words, and that they did this more often than did English speakers. English speakers appeared to mispronounce tones on words across the board, and would more often than Mandarin speakers incorrectly name pictures that belonged to words with contour tones as words with level tones. For instance, whereas English speakers would occasionally make rise-to-low and fall-to-low errors in picture-naming, Mandarin speakers never made such errors (Figure 7).

The observation that tone type did not strongly determine English speakers’ performance while it did strongly determine Mandarin speakers’ performance converges with previous accounts from the literature. On one hand, English speakers—and non-tonal L1 speakers in general—may be relatively unaffected by potential interference from native intonational categories. This enables them to process non-native tones in a psychoacoustic way (Best, 2019, p. 5; K. Yu et al., 2019), which would result in relatively uniform performance in non-native tone processing regardless of the tone type. On the other hand, Mandarin speakers may process tones in a non-native system through the lens of their native tone categories. In the case of the non-native tone system employed in our tasks, this would lead to a relative difficulty in the distinction of level tones, given that these tone types are hypothetically problematic for Mandarin speakers both in phonetic-acoustic and phonological-categorical terms (Francis et al., 2008; Qin & Jongman, 2016). Although these accounts all stem from the perception literature, it appears this account is also valid for production. Indeed, based on their findings of non-native Cantonese tone perception and production, A. Yu et al. (2022, p. 20) suggest that an L1 intonational system “might not exert as strong an effect on L2 tonal acquisition” as an L1 tone system. Our production data support this view.

### 5.3 Contribution of extralinguistic factors

Finally, in RQ3 we probed the extent to which musical experience, pitch aptitude, and WM facilitated phonetic and phono-lexical production accuracy of non-native tones.

Musical experience did not enhance phonetic accuracy in the imitation task. This was against the hypothesis that musical experience would facilitate tone imitation, based on earlier studies that showed that musicianship (defined either by years of practice or by musicality tests) led to more native-like imitations of Mandarin tones by English-L1 speakers (Gottfried et al., 2004; Li & Dekeyser, 2017). Methodological differences (in terms of the measure of musicianship and imitation accuracy) may in part explain the discrepancy between previous findings and those in our study. An additional explanation for the lack of a facilitative effect in our study could be that imitation is a relatively undemanding and potentially easy task (Hao & de Jong, 2016), in which performance may not be strongly facilitated by extralinguistic factors. Indeed, musical experience did facilitate phono-lexical accuracy in the more cognitively demanding picture-naming task. This is in line with our prediction, and coincides with findings from earlier studies on tone word learning in the listening modality (Cooper & Wang, 2012; Laméris & Post, 2022). However, Laméris and Post (2022), which involved the same participants as in the present study, found that musical experience particularly facilitated English, but not Mandarin speakers’ performance in tone word learning. The interpretation of this apparent differential in relevance of musical experience was that the combined effect of pitch acuity gained from either linguistic or musical experience may not be additive, cf. Cooper and Wang (2012). The results from the present study, which examined tone word learning in the speaking modality, do not clearly show that Mandarin speakers benefited less from musical experience in comparison to English speakers.^4^ Previous studies (Chang et al., 2016, p. 2446; Qin et al., 2021, p. 436) have suggested that the strength of the effect of musicianship on tone processing depends heavily on the measure of musicianship, the tonal stimuli involved, and the level of processing that a task involves (pre-lexical or lexical). It may be that in addition, the facilitative effect of musicianship also depends on the modality that a task involves (listening or speaking).

There was no conclusive evidence that individual pitch perception aptitude enhanced phonetic accuracy in imitation. The tonal distance model only suggested that pitch aptitude led to more target-like productions for Mandarin speakers’ imitations of falling tones on Day 2. Counterintuitively, there was also evidence that pitch aptitude was associated with less accurate imitations of mid-level tones for the Mandarin group on Day 2. Overall, we found no convincing facilitative effect of individual pitch aptitude on phonetic accuracy in tone imitation, cf. Hao (2012), but cf. Dong et al. (2019). One explanation for the lack of a clear facilitative effect of pitch perception aptitude in the present study could be that perceptual skills are not necessarily strongly indicative of production skills, as has been proposed by the “skill-specificity hypothesis” (Li & Dekeyser, 2017). Another explanation—which is similar to our explanation as to why tone imitation was not facilitated by musical experience—is that tone imitation may be a relatively easy task for which any differences in individual performance are not strongly determined by extralinguistic resources.

Indeed, we found that for the more cognitively demanding picture-naming task, pitch aptitude was in fact facilitative. This chimes in with perceptual studies that show that the ability to process tones pre-lexically is a steppingstone for lexical processing (Laméris, 2022; Laméris & Post, 2022; Ling & Grüter, 2022; Wong & Perrachione, 2007). Our findings suggest that this link between pre-lexical and lexical processing is maintained even when the pre-lexical and lexical tasks at hand involve processing in a different modality. Indeed, there were striking parallels between performance in the tone categorization task that measured pitch aptitude (Laméris & Post, 2022) and the present study’s picture-naming task. Most notably, in both tasks Mandarin speakers performed poorly on the processing of the low-level tones.

Our results only weakly suggested a facilitative effect of WM on phonetic and phono-lexical production of tones. Yet, the observed direction of the effect points toward what we had predicted. That is, individuals with higher backwards digit span scores appeared to have more target-like imitation and higher likelihoods of correct picture-naming. Although we are cautious to derive strong conclusions from these findings, they tentatively support the notion that the ability to recall digit sequences is linked to the ability to accurately listen and repeat sound sequences, and to use those sound sequences at a lexical level (Gupta, 2003). We note that the effect of WM on non-native tone processing may heavily depend on the measure of WM and the task at hand (Bidelman et al., 2013; Hutka et al., 2015), but at the very least our findings suggest that WM capacity can explain some degree of individual variability in non-native tone processing in the speaking modality.

### 5.4 Final considerations

Some limitations to the current study must be acknowledged. First, the use of pseudoword tones, which were only manipulated for F0, may limit its applicability to real-life tone learning. However, the use of pseudowords allowed us to directly compare a group of tonal and non-tonal L1ers and investigate the effect of L1 tonal status and tone type (contrasting in contour and in level) on *ab initio* production in a novel tone system, unlike previous cross-linguistic production studies in which participants had prior knowledge of the target language (Hao, 2012; Yang, 2019; A. Yu et al., 2022).

Second, the participants’ L1s (English and Mandarin) are among the most researched languages in the tone learning literature (if not the most researched), so to make more general claims about the potential effect of L1 tonal status on the ability to learn and produce tones in an L2, speakers from other languages need to be included in future studies.

Finally, it is worth considering that the present production study largely replicates findings from the perception literature. This may raise the question of whether the production data reported in this study have any meaningful addition to what we already know from perception. In particular, a comparison with the perceptual counterpart of this study (Laméris & Post, 2022) reveals strong parallels in terms of performance per tone type and in terms of the effects of language-specific and extralinguistic factors on performance. If we assume that within the microcosm of this experiment, participants had reached the later stages of speech learning on Day 2 of the perception and production tasks, this remarkable similarity between the listening and speaking modalities confirms both previous empirical findings (Zhang & Peng, 2017) as well as theoretical predictions proposed by the (revised) Speech Learning Model that perceptual performance converges (Flege, 1995) or co-evolves (Flege & Bohn, 2021) with productive performance over time. Although the perception-production link was not the focus of this article, it may be that studies from the listening modality can in fact tell us a lot about what may happen in the speaking modality. Yet, we still find value in studying the production side of speech processing in addition to the perception side. This is not least because tone production studies in the tone learning literature are still relatively scarce, and there is thus still much left to explore, but also because production is quintessentially different from perception, regardless of how strongly the two modalities may be linked (Baese-Berk, 2019; Flege & Bohn, 2021; Schmitz et al., 2018).

## 6 Conclusion

We investigated the effects of L1 tonal status, tone type, and extralinguistic factors on non-native tone learning facility in the speaking modality. The results from an imitation and picture-naming task revealed no clear facilitative effect of L1 tonal status. Mandarin participants did not outperform English speakers in tone production in a non-native tone system. Instead, tone production accuracy in both tasks was mostly determined by the specific tone types, which were produced with various degrees of accuracy depending on participants’ L1. In particular, Mandarin speakers appeared to struggle with level tone contrasts. In imitation, they tended to produce low-level tones with a relatively high pitch compared with English speakers. In picture-naming, they frequently mispronounced low-level tone words as mid-level tone words, and did so much more often than did English speakers. English speakers appeared to be less influenced by tone type in both the imitation and picture-naming tasks. Performance in both tasks was further modulated by individual extralinguistic resources (musical experience, pitch aptitude, and WM), albeit to different degrees.

Production data are still relatively scarce in the tone learning literature, and previous cross-linguistic tone production studies did not investigate production in a system that was non-native to all participants involved (Ding et al., 2011; Kirby & Giang, 2021; Y. Wang et al., 2003) or investigated production by non-native speakers who had prior knowledge of the target language (Hao, 2012; Yang, 2019; A. Yu et al., 2022). This leaves it relatively unclear what the driving factors are behind individual differences in tone production at the earliest stages of encountering a non-native tone system. The present study, in which both non-tonal and tonal L1ers learned non-native pseudowords, and in which individual measures of musical experience, pitch aptitude, and WM were accounted for, suggests that an individual’s phonetic and phono-lexical accuracy of non-native tone production is primarily determined by the tone type to be produced (and the potential interaction with native tone types), and further facilitated by individual extralinguistic resources.

## Supplemental Material

sj-docx-1-las-10.1177_00238309221143719 – Supplemental material for Phonetic and Phono-Lexical Accuracy of Non-Native Tone Production by English-L1 and Mandarin-L1 Speakers Click here for additional data file. Supplemental material, sj-docx-1-las-10.1177_00238309221143719 for Phonetic and Phono-Lexical Accuracy of Non-Native Tone Production by English-L1 and Mandarin-L1 Speakers by Tim Joris Laméris, Katrina Kechun Li and Brechtje Post in Language and Speech

sj-docx-2-las-10.1177_00238309221143719 – Supplemental material for Phonetic and Phono-Lexical Accuracy of Non-Native Tone Production by English-L1 and Mandarin-L1 Speakers Click here for additional data file. Supplemental material, sj-docx-2-las-10.1177_00238309221143719 for Phonetic and Phono-Lexical Accuracy of Non-Native Tone Production by English-L1 and Mandarin-L1 Speakers by Tim Joris Laméris, Katrina Kechun Li and Brechtje Post in Language and Speech

## Appendix A

**Table A1.** Detailed Participant Demographics (English Group).

ID	Age	L2s and self-reported level (0–10)	Currently Practicing	ME	WM	PP
EN-MU-F-1	21	German 3	Keyboard/Piano; Woodwind; Singing	14	47	100
EN-MU-F-2	19	Spanish 7, Portuguese 6	Drums; Keyboard/Piano; Singing	14	27	100
EN-MU-F-3	20	–	Keyboard/Piano; Strings; Woodwind	14	74	100
EN-MU-F-4	19	French 5, Spanish 4	Woodwind; Choral Singing	11	62	99
EN-MU-F-5	20	–	Guitar; Choral Singing; Singing	12	31	100
EN-MU-F-6	20	Gujarati^a^ 5, Spanish 4, French 1	Keyboard/Piano; Strings; Choral Singing	13	71	100
EN-MU-M-1	20	–	Strings; Singing	13	61	99
EN-MU-M-2	20	–	Keyboard/Piano; Strings; Brass; Choral Singing	16	100	100
EN-MU-M-3	20	–	Keyboard/Piano; Woodwind; Choral Singing	12	81	96
EN-MU-M-4	25	Russian^a^ 7, French 7, German 7, Spanish 7	Keyboard/Piano	19	91	97
EN-MU-M-5	22	Italian^a^ 7, Spanish 4, French 1	Guitar; Keyboard/Piano; Singing	8	62	100
EN-NM-F-1	19	French 7, Spanish 2	–	–	17	76
EN-NM-F-2	22	French 5, Hindi 3	–	2	42	86
EN-NM-F-3	23	Spanish 4	–	–	53	65
EN-NM-F-4	21	–	–	–	65	94
EN-NM-F-5	21	Spanish 3	–	–	29	99
EN-NM-M-1	21	German 7	–	5	97	86
EN-NM-M-2	20	Hindi^a^ 7	–	–	44	56
EN-NM-M-3	23	German 6	–	2	63	63
EN-NM-M-4	22	–	–	–	37	92
EN-NM-M-5	22	–	–	–	62	78

ME: musical experience (years); WM: working memory score (0–100); PP: pitch perception aptitude score (0–100).

a Exposure to a (heritage) language before the age of 12 at home or in other surroundings.

## Appendix B

**Table B1.** Detailed Participant Demographics (Mandarin Group).

ID	Age	L2s and self-reported level (0–10)	Currently practicing	ME	WM	PP
MA-MU-F-1	20	English 8, Wu^a^ 7, Japanese 6	Keyboard/Piano; Woodwind; Choral Singing	16	55	99
MA-MU-F-2	20	English 8, Italian 1, French 1	Strings	17	92	90
MA-MU-F-3	19	English 6	Guitar; Keyboard/Piano; Singing; Guzheng	17	62	97
MA-MU-F-4	29	English 8, Cantonese^a^ 7, French 2	Guzheng	22	31	82
MA-MU-F-5	24	English 8, Cantonese^a^ 7, French 1	Keyboard/Piano	14	90	99
MA-MU-M-1	24	English 10, French 1	Erhu	18	93	99
MA-MU-M-2	23	English 8, Cantonese^a^ 7	Guitar	8	52	96
MA-MU-M-3	28	English 8, Wu^a^ 7, German 5, Cantonese 2	Keyboard/Piano; Strings; Choral Singing	15	31	94
MA-MU-M-4	19	English 8	Strings; Singing	9	30	100
MA-MU-M-5	19	English 8, French 1	Guitar; Keyboard/Piano	12	91	97
MA-NM-F-1	29	Italian 10, English 8, Wu^a^ 7, French 7, Japanese 2, Persian 2	–	3	87	99
MA-NM-F-2	23	English 8	–	2	95	90
MA-NM-F-3	25	English 8	–	–	41	97
MA-NM-F-4	22	English 8	–	–	92	97
MA-NM-F-5	24	English 8, Japanese 7	–	–	79	99
MA-NM-M-1	18	English 7	–	1	77	94
MA-NM-M-2	19	English 8	–	1	94	99
MA-NM-M-3	20	English 8	–	1	83	100
MA-NM-M-4	23	English 8	–	2	91	92
MA-NM-M-5	23	Kunming Chinese^a^ 7, English 7	–	–	75	86

ME: musical experience (years); WM: working memory score (0–100); PP: pitch perception aptitude score (0–100).

a Exposure to a (heritage) language before the age of 12 at home or in other surroundings.^b^

b It is noted that some of the Chinese L2s reported by the Mandarin speakers have level tone contrasts unlike Mandarin, which may have affected performance on the mid- and low-level tones. However, a visual inspection of performance in the picture-naming task by participants who reported a L2 with level tone contrasts versus participants who did not, did not reveal notable differences (see Supplementary Material). In addition to the fact that all participants reported that Mandarin was their L1 and the language they used the most, it was therefore deemed fit to group these participants together.

## Notes

1. In the present discussion, we use the term “L2” to refer to any non-native language, regardless of the order in which that language was acquired in addition to an individual’s native language.

2. We refrain from using the word “evidence” in our interpretation of parameter estimate, because the posterior distribution quantifies our uncertainty about an estimate of an effect, and the use of the terms “evidence,” “weak evidence,” and “no evidence” may be oversimplifications, as pointed out by Nicenboim et al. (2018, p. 1079).

3. Although the development of phonetic accuracy over time was not a main factor of interest, the fact that the imitation task was conducted on 2 days allowed us to see if there were any differences in imitation accuracy, either between L1s or tone types on a specific day, or between days for a specific L1 or tone type.

4. We exploratorily investigated whether the effect of musical experience on picture-naming differed between English and Mandarin speakers. Multiple comparisons revealed that the estimate size for the effect of musical experience was in fact larger for English speakers, *b* = 2.80 (0.02, 6.30) than for Mandarin speakers, *b* = 1.28 (−0.80, 3.80). However, this finding should be interpreted with caution, given that we found no clear suggestion for an interaction between L1 and musical experience in the present model, *b* = 0.77, (−1.14, 2.74), *pd* = 81.18%.
