# Supplementary material for: Phonetic and Phono-Lexical Accuracy of Non-Native Tone Production by English-L1 and Mandarin-L1 Speakers
Source: Lang Speech. 2023 Jan 15;66(4):974–1006. doi: 10.1177/00238309221143719 (PMC10666469; doi:10.1177/00238309221143719)
Supplement: sj-docx-1-las-10.1177_00238309221143719 – Supplemental material for Phonetic and Phono-Lexical Accuracy of Non-Native Tone Production by English-L1 and Mandarin-L1 Speakers [file sj-docx-1-las-10.1177_00238309221143719.docx]

**Supplementary Material: Details on Working Memory and Pitch Aptitude Pre-Tests**
This supplementary material contains more detailed information on the Working Memory and Pitch Aptitude Pre-Tests that are mentioned in the main manuscript.  **1 Working Memory (WM) Pre-Test**

Participants were instructed to repeat out loud in their native language and in backward order a sequence of digits presented to them on the screen. After a practice session, they were presented with a block of five 2-digit sequences (e.g. 1-7; 6-3; 2-5; 8-4; 9;5). Participants would move onto a next block of five n+1-digit sequences (e.g. 5-8-2; 6-9-4; etc.) and continue to do so if they correctly repeated at least three sequences per block. If participants did not reach this threshold, the task was aborted at the end of a block. The maximum attainable block consisted of five 8-digit sequences.
 A percental working memory score was calculated by dividing the total number of digits from fully correctly recalled sequences by the maximum attainable score (175). **2 Pitch Perception Aptitude Pre-Test**

***2.1 Stimuli***

A set of vowels (/i/ /a/ and /ɛ/) with each four pitch patterns (rise, fall, mid-level and low-level), resulting in a total of 9 unique stimuli were used for the pitch perception aptitude pre-test. These vowels were recorded by a female Italian speaker and stimuli were generated in the same way as described in the main manuscript. It is worth noting that the tones in the pitch aptitude pre-tests had the same relative tone values in terms of Chao numerals as the pseudolanguage word stimuli used in the imitation task. They were therefore deemed to belong to the same four tone categories: namely 15 (Rise); 51 (Fall); 22 (Mid-level); and 11 (Low-level). For visualization, the f_0_ and Chao-normalized contours of the pre-test tones are shown in Figure 1.

***2.2 Procedure*** Participants listened to a vowel carrying one of the four tones and were asked to identify the tone by touching the corresponding arrow on the touchscreen (Figure 2). They were encouraged to make their choice as quickly as possible and to guess if unsure. Time-out was 5000 ms after presentation of the audio stimulus.

One practice session with 16 trials (4 presentations per tone) including feedback was held at the beginning. In the practice session, the vowel /o/ was used, which was not used in the main session. The practice session was followed by a main session in which there were 72 trials (6 presentations per stimulus) without feedback in a randomized order.

**Figure 1:** f0 and Chao numeral curves for tones employed in the Pitch Perception Pre-Test (Female, Vowels) and in the Imitation Task (Male, Words).


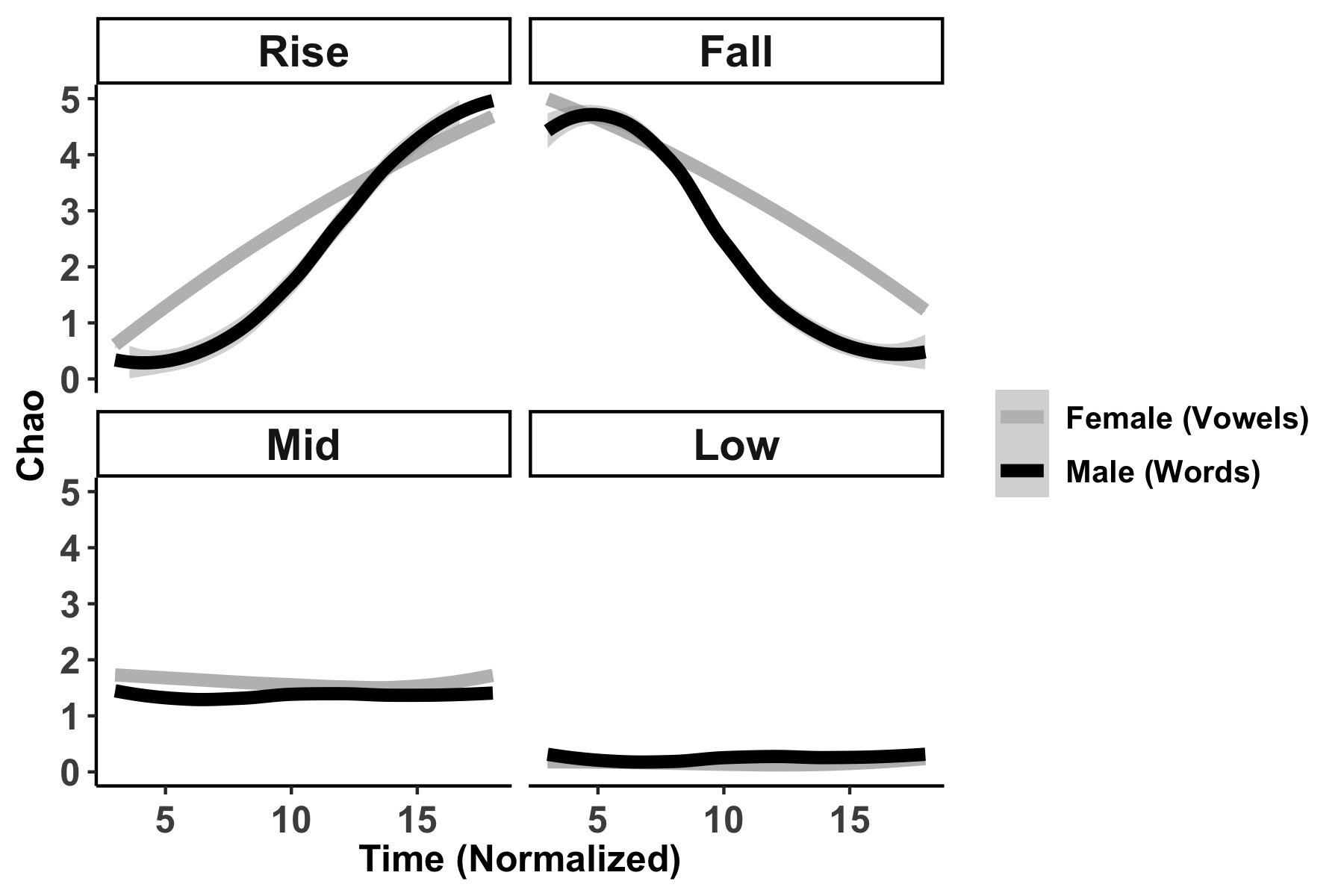

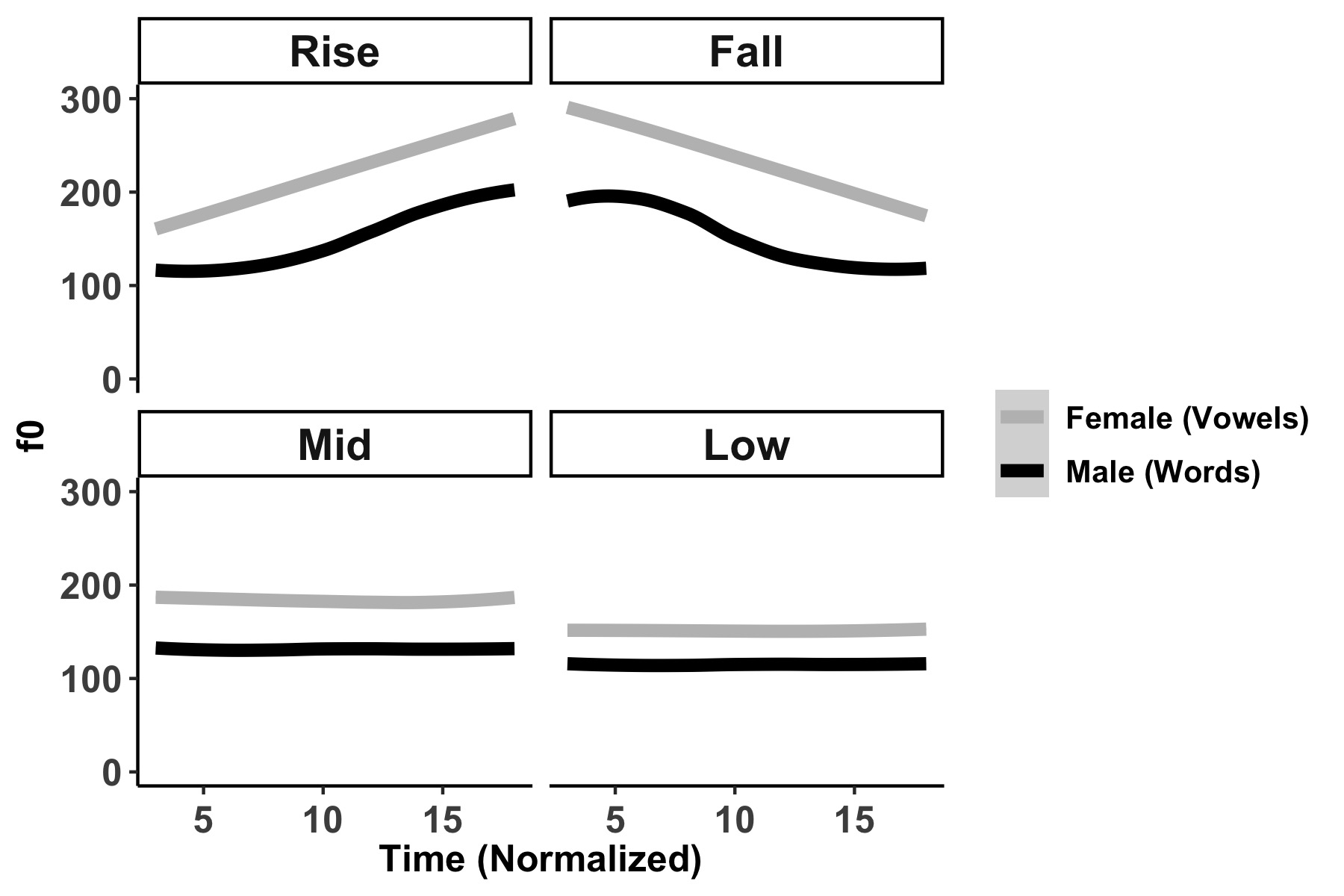


**Figure 2:** Visual stimuli in Tone Categorization Task


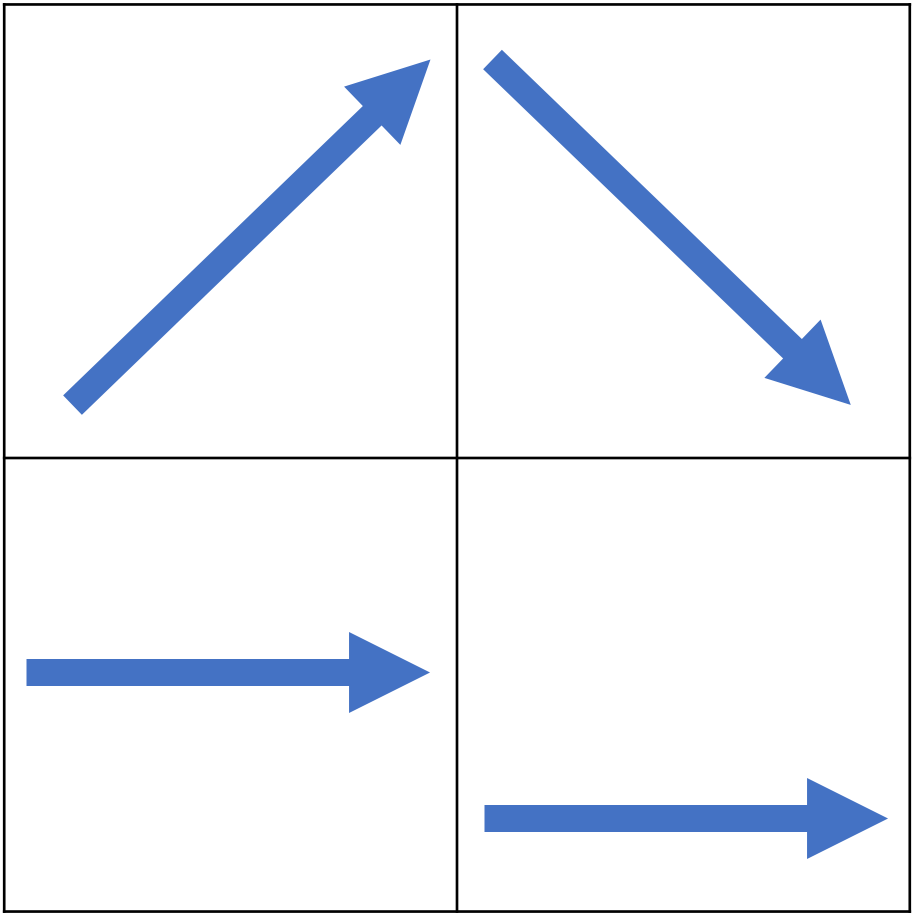


***3. Results***

Equivalence tests (Lakens et al., 2018) with Cohen’s d set at 0.5 revealed no significant differences in the measures between the two groups.

Test results
Age: t(39) = -0.453, p = 0.673
WM: t(39) = -0.344, p = 0.634
Pitch Perception Aptitude t(39) = -0.048, p = 0.519
Musical Experience (Musicians) t(19) = 0.288, p = 0.388
Musical Experience (Non-Musicians) t(18) = 0.986, p = 0.168

In addition, following Dong et al. (2019), a linear mixed-effects model on accuracy (dependent variable : correct/incorrect) was run with a fixed effect of *L1* (English, Mandarin, reference = English) and random intercepts for *subject* and *item.* This revealed no significant difference in accuracy likelihood in pitch perception aptitude between the two groups (*b* = 0.32, *SE* = 0.54, *p* = 0.548).


***References***

Dong, H., Clayards, M., Brown, H., & Wonnacott, E. (2019). The effects of high versus low talker variability and individual aptitude on phonetic training of Mandarin lexical tones. *PeerJ*, *7*(8), e7191. https://doi.org/10.7717/peerj.7191

Lakens, D., Scheel, A. M., & Isager, P. M. (2018). Equivalence Testing for Psychological Research: A Tutorial. *Advances in Methods and Practices in Psychological Science*, *1*(2), 259–269. https://doi.org/10.1177/2515245918770963
