# Supplementary material for: Phonetic and Phono-Lexical Accuracy of Non-Native Tone Production by English-L1 and Mandarin-L1 Speakers
Source: Lang Speech. 2023 Jan 15;66(4):974–1006. doi: 10.1177/00238309221143719 (PMC10666469; doi:10.1177/00238309221143719)

**Supplementary Material: Details on performance by Mandarin participants with knowledge of other Chinese languages.**
It is noted that some (varieties) of the Chinese L2s (Cantonese, Wu, Kunming Chinese) reported by the Mandarin speakers have level tone contrasts unlike Mandarin, which may have affected performance on the mid- and low-level tones. However, a visual inspection of performance and error types in the pitch aptitude pre-test (“Tone Categorization”) and the picture-naming task (“Word Production”) by participants who reported a L2 with level tone contrasts (“Level Dialects”, in Blue) versus participants who did not (“No Level Dialect”, in Red), did not reveal notable differences. In addition to the fact that all participants reported that Mandarin was their L1 and the language they used the most, it was therefore deemed fit to group these participants together.

***Supplementary Figure 1:*** *Performance for Level and Non-Level L2 participants*


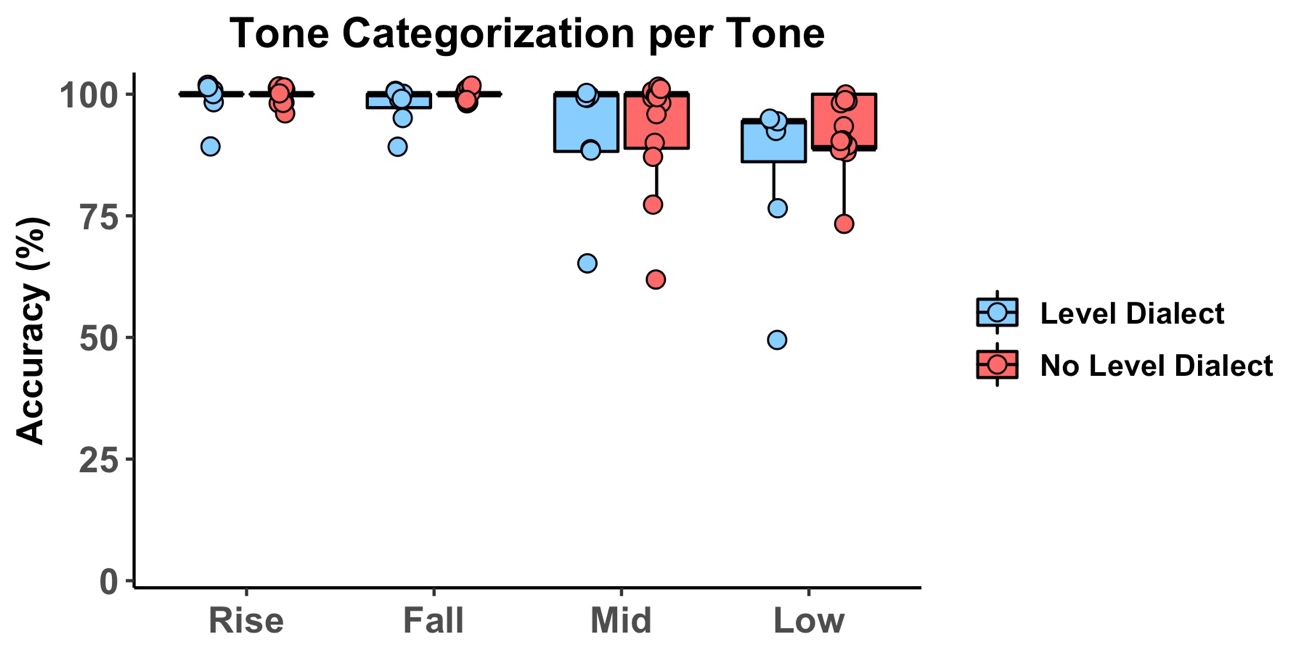

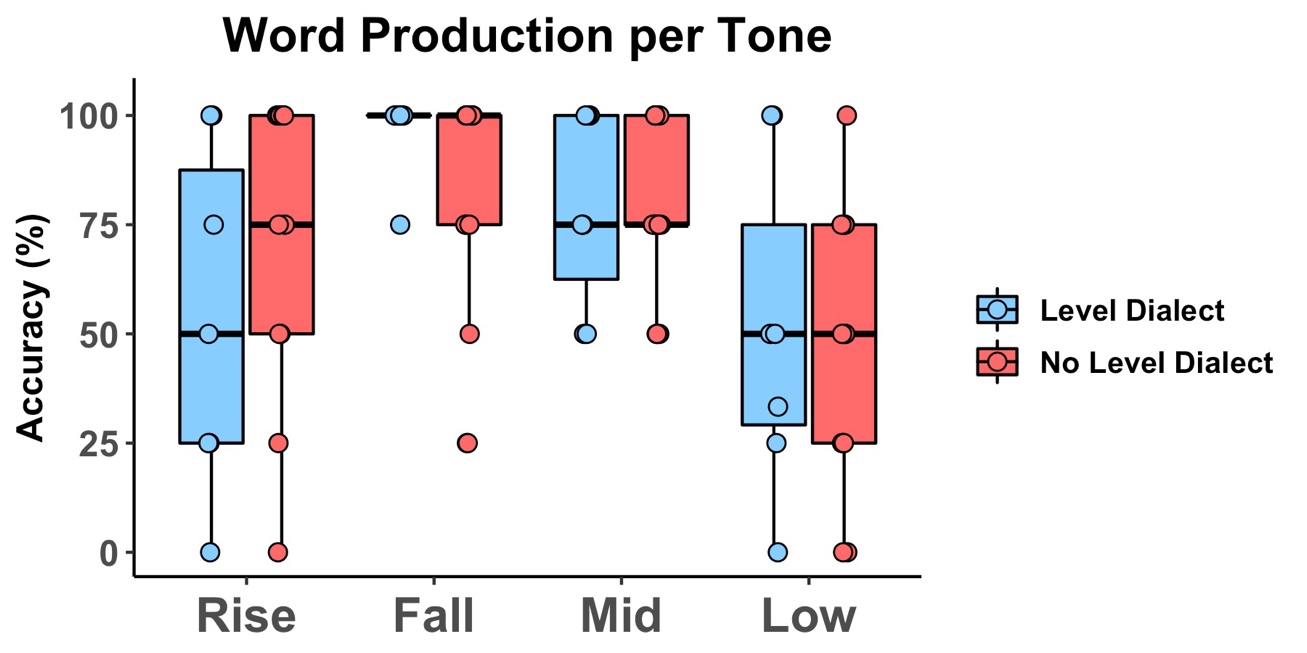


***Supplementary Figure 2:*** *Error types for Level and Non-level L2 participants*


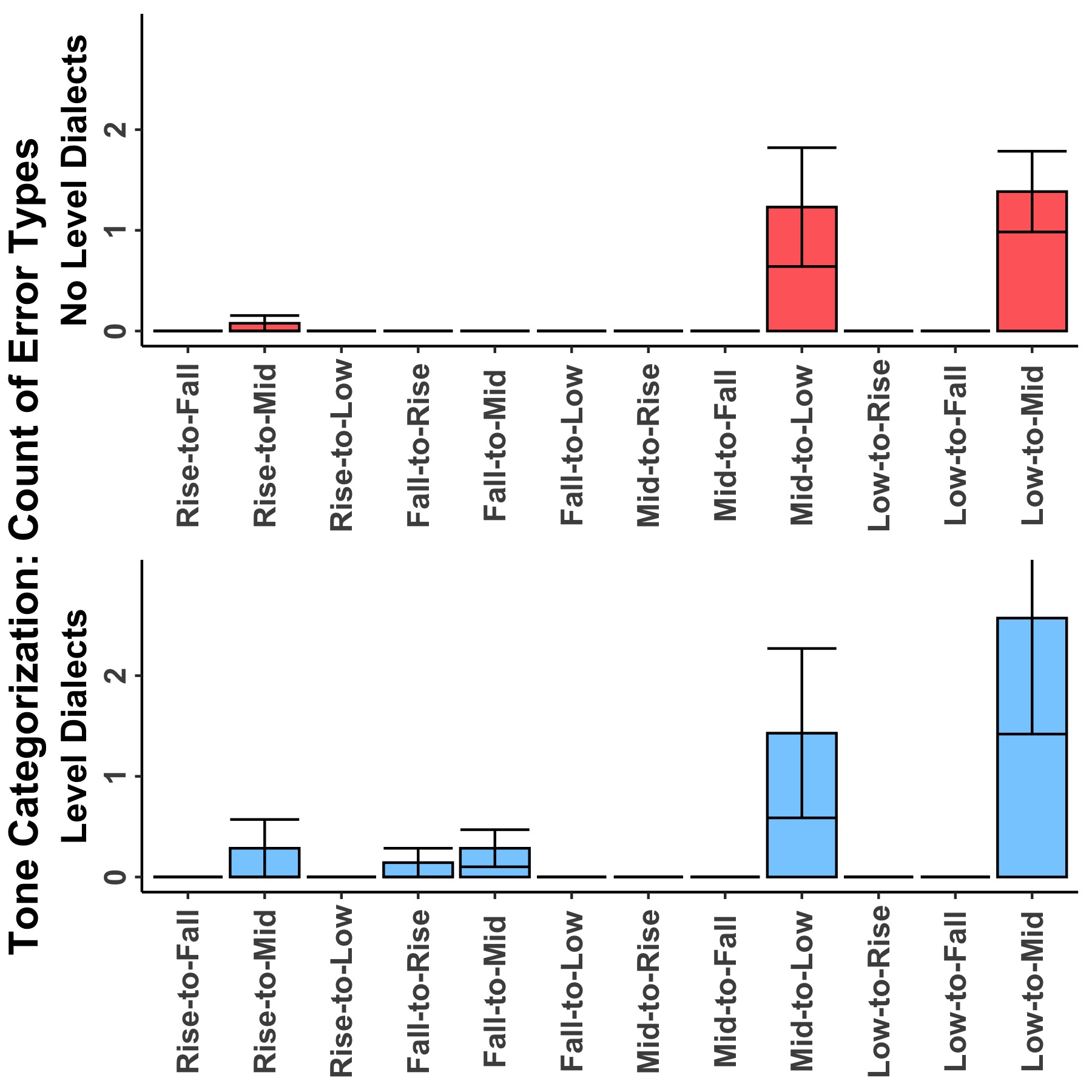

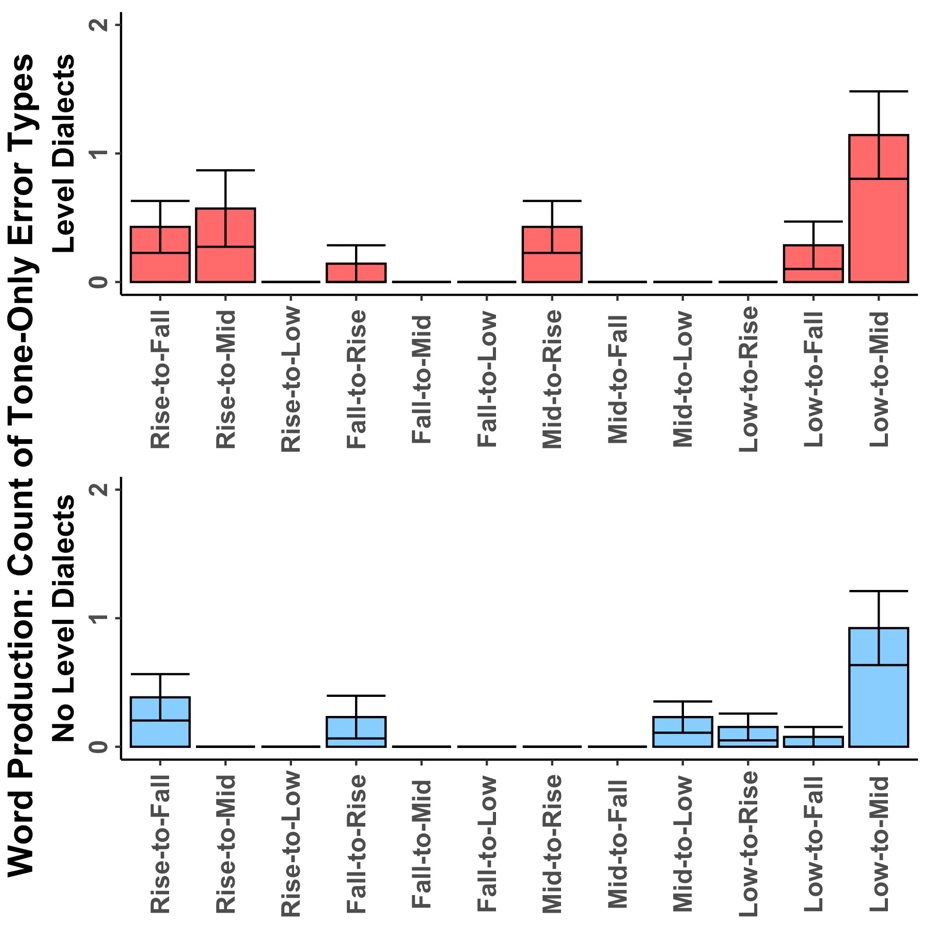

Supplement: sj-docx-2-las-10.1177_00238309221143719 – Supplemental material for Phonetic and Phono-Lexical Accuracy of Non-Native Tone Production by English-L1 and Mandarin-L1 Speakers [file sj-docx-2-las-10.1177_00238309221143719.docx]
